# Premonitory urge in tic disorders – a scoping review

**DOI:** 10.3389/fpsyt.2025.1504442

**Published:** 2025-01-30

**Authors:** John B. Wohlgemuth, Kelly H. Watson, Kayce D. Gill, David A. Isaacs

**Affiliations:** ^1^ Department of Neurology, Icahn School of Medicine at Mount Sinai, New York City, NY, United States; ^2^ Department of Neurology, Vanderbilt University Medical Center, Nashville, TN, United States; ^3^ Annette and Irwin Eskind Family Biomedical Library and Learning Center, Vanderbilt University, Nashville, TN, United States

**Keywords:** Tourette syndrome, tic disorder, tic, premonitory urge, premonitory sensation, sensory

## Abstract

**Introduction:**

Premonitory urges are uncomfortable bodily sensations preceding tics. They are highly prevalent, frequently bothersome, and increasingly recognized as a central phenotypic feature in tic disorder populations. This scoping review aimed to systematically consolidate published knowledge and identify knowledge gaps regarding premonitory urges in primary tic disorders.

**Methods:**

Search strategies were deployed in five databases and five topic-relevant journals. Two independent reviewers screened all candidate abstracts against predefined inclusion criteria. One hundred and fifty-five articles were included in the scoping review. The same two reviewers independently extracted and consolidated pertinent data from included articles.

**Results:**

Multiple methods for assessing premonitory urge were identified, each with strengths and weaknesses. The subjective quality of premonitory urges varies between individuals, with increased prevalence of a “not just right” urge quality in individuals with comorbid obsessive-compulsive disorder. Awareness of premonitory urge appears to arise several years after tic-onset, yet many individuals perceive their tics as voluntary responses to premonitory urges. Premonitory urges and tics are temporally coupled in real time, but premonitory urge severity and tic severity, as assessed by clinical scales, are not consistently associated. The mechanistic and developmental relationship between premonitory urges and tics remains unclear. Data are limited on premonitory urge response to treatment, but several promising interventions were identified. The insula and supplementary motor area are the neuroanatomical structures most strongly implicated in emergence of the premonitory urge.

**Discussion:**

Knowledge of the clinical characteristics, measurement, and neural mechanisms of premonitory urge has advanced considerably in recent years, but important knowledge gaps remain in each of these domains. Addressing these knowledge gaps will be key to developing effective interventions for premonitory urge.

**Systematic Review Registration:**

Open Science Framework (OSF) https://doi.org/10.17605/OSF.IO/WT43Z.

## 1 Introduction

Primary tic disorders (1), including Tourette syndrome (TS), chronic motor tic disorder, chronic vocal tic disorder, and transient tic disorder, are estimated to affect 0.77%, 1.65%, 0.69%, and 2.99% of the global population, respectively (2). Tics are the defining feature of these disorders, but the vast majority of individuals in these populations also experience premonitory urges. Premonitory urges are uncomfortable bodily sensations preceding tics, often building in the moments before a tic and resolving once the tic is performed. In Cohen and Leckman’s seminal 1992 study of sensory phenomena in TS, nearly 80% of participants endorsed premonitory urges, and nearly 60% reported their premonitory urges were more distressing than tics themselves (3). Subsequent research in other tic disorder samples has replicated these original findings, demonstrating that premonitory urges are common, particularly in adolescence and adulthood, and bothersome.

Since the initial publications describing premonitory urges more than thirty years ago, numerous studies have sought to characterize them, clarify their relationship with tics, identify their clinical and neurobiological correlates, and, to a lesser extent, treat them. As a result of these research endeavors, knowledge of premonitory urges has significantly advanced over the past three decades. However, given the complexity of the phenomenon, as well as the diverse methods employed and populations recruited to study it, much ambiguity remains concerning premonitory urges. Critically, the developmental and pathophysiologic interrelationship between premonitory urges and tics is unclear, and an effective treatment for premonitory urges has yet to be established. A comprehensive review of published research is needed to consolidate the breadth of current knowledge about premonitory urges and, equally important, to identify limitations and gaps in this knowledge. Reviews of the premonitory urge literature have previously been conducted but have focused on specific aspects of the phenomenon (4, 5) (e.g., neurobiological correlates) or adopted a non-systematic approach (6, 7). To address these limitations, we conducted a scoping review of premonitory urge in primary tic disorders.

## 2 Methods

This scoping review was conducted in accordance with the Joanna Briggs Institute (JBI) methodology for scoping reviews (8). The study protocol was registered on Open Science Framework (OSF) (https://osf.io/b6tnf/files/osfstorage/65b18bb94aa63c067bdf2550). To be eligible for inclusion, studies had to be original research, published in English, and contain information about premonitory urge in primary tic disorders. There was no restriction based on study date. Studies involving participants with functional tic-like behaviors (9) were not included unless participants with primary tic disorders were also enrolled; for such studies, only data on individuals with primary tic disorders were reviewed and extracted. This scoping review considered experimental and quasi-experimental studies (i.e., randomized controlled trials, non-randomized controlled trials, before and after studies, and interrupted time-series studies), analytical observational studies (i.e., prospective and retrospective cohort studies, case-control studies, and analytical cross-sectional studies), descriptive observational studies (i.e., case series, individual case reports, and descriptive cross-sectional studies), and qualitative studies. Conference abstracts, grey literature, unpublished works, literature reviews of any type, and opinion papers were excluded.

**Figure 1:**
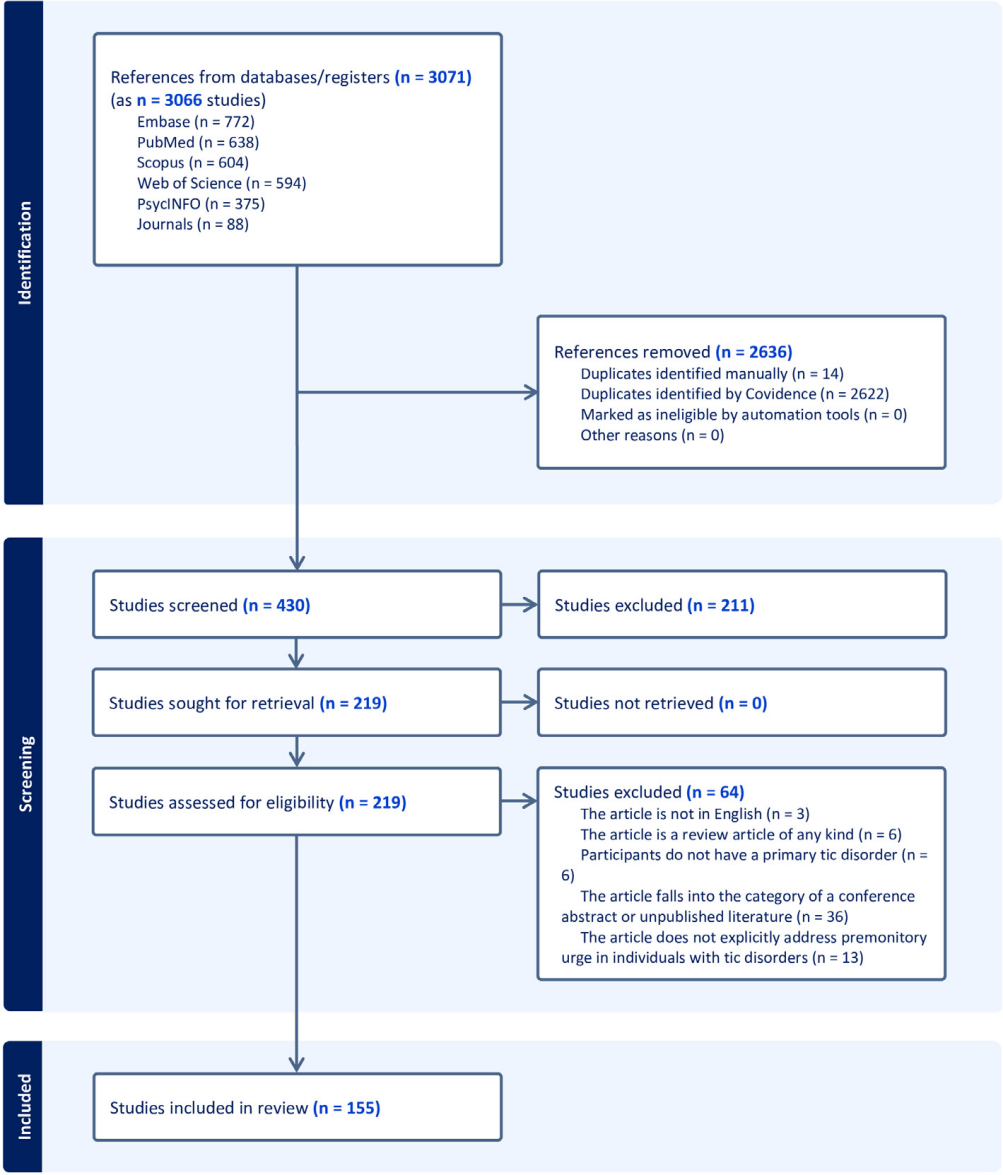
Premonitory urge in tic disorders.

PubMed (National Library of Medicine), Embase (Elsevier), PsycINFO (ProQuest), Web of Science (Clarivate), and Scopus (Elsevier) were searched for relevant articles. The journals Parkinsonism and Related Disorders, CNS Spectrums, Cognitive Neuropsychiatry, Behavioral Neurology, and Cognitive and Behavioral Neurology were also independently searched as they are not fully indexed in the included databases. A biomedical librarian (K.D.G.) developed the search strategy consisting of a combination of relevant keywords and database-specific subject terms. The search was conducted on January 9, 2023, and a revised search was conducted on January 24, 2024 after the authors identified studies not captured by the original search. The finalized search strategies are available in the **Supplementary Material**. Following the search, all identified citations were imported into Covidence (10), a systematic review management software. Covidence was also used to deduplicate results from the revised searched. Titles and abstracts of all imported publications were independently evaluated for inclusion or exclusion by two reviewers (D.A.I. and J.B.W.). Subsequently, the same two reviewers (D.A.I. and J.B.W.) independently assessed the full text of the included publications using the predefined inclusion criteria. Inter-reviewer disagreements at each stage of the selection process were resolved by the independent decision of a third reviewer (K.H.W.). Results of the search and study inclusion process are presented in a Preferred Reporting Items for Systematic Reviews and Meta-analyses extension for scoping review (PRISMA-ScR) flow diagram (**Figure 1**) (11).

Data were extracted by two independent reviewers (D.A.I. and J.B.W.) using a data extraction tool (presented in the **Supplementary Material**) developed by the reviewers. The following data, if relevant and available, were extracted from eligible articles: 1) clinical characteristics of the study sample; 2) method of premonitory urge assessment; 3) qualitative and/or quantitative characteristics of premonitory urge; 4) associations between premonitory urge characteristics and other clinical characteristics; 5) premonitory urge response to treatment and/or tic suppression; and 6) neural correlates of premonitory urge and method for investigating such neural correlates. Within each of the above categories, additional specific data elements were extracted (as shown in the **Supplementary Material**). Following data extraction for all studies, the two reviewers consolidated their findings and arrived at a consensus as to the relevant data to be included from each article. All relevant findings were grouped into one of the above six categories. The overarching term “tic disorder” is used in this review to refer to all primary tic disorders including TS, chronic (persistent) motor tic disorder, chronic (persistent) vocal tic disorder, and transient tic disorder, but not functional tic disorder. The term “CTD” is used to refer collectively to TS, chronic motor tic disorder, and chronic vocal tic disorder.

## 3 Results

### 3.1 Methods of premonitory urge ascertainment

Of the 155 studies meeting inclusion criteria, 25 examined methods to measure premonitory urge (**Table 1** contains descriptions of assessment methods). The majority of these studies employed questionnaire-based methods, but a small minority employed urge monitors.

**Table 1:** Methods of premonitory urge measurement.

Name of Assessment	Initial Publication Proposing the Assessment	Type of Assessment	Description of Assessment	Notes/Comments
Premonitory Urge for Tics Scale (PUTS)	Woods et al. (2005) (12)	Self-report scale – no reference to a specific timeframe	The first 9 items are statements about the sensations preceding tics; the tenth item is a statement about tic suppression ability. Respondents endorse each item on a Likert scale from 1 (“not at all true”) to 4 (“very much’’). The tenth item is a statement about tic suppression ability. The scale score is the sum of the individual item scores.	The tenth item is often excluded, resulting in a 9-item scale with scores ranging from 9 (absent/minimally intense) to 36 (maximally intense).
Premonitory Urges for Tic Disorders Scale-Revised (PUTS-R)	Baumung et al. (2021) (14)	Self-report scale – no reference to a specific timeframe	The proposed changes to the PUTS included, among others, rephrasing items 1 and 9, adding items to the scale, creating an urge severity and urge quality subscale, and changing the response options from a 4-point to a 5-point Likert scale.	No studies to date have examined the psychometric properties of the PUTS-R.
Individualized Premonitory Urge for Tics Scale (I-PUTS)	McGuire et al. (2016) (33)	Clinician-administered scale - assesses premonitory urge symptoms over the past week	The scale parallels the Yale Global Tic Severity Scale (YGTSS) tic inventory. For each reported tic the presence of an associated urge is determined. If present, the clinician then assesses urge frequency on a 4-point Likert scale (1 = “Urge occurs 0-25% of the time you do the tic” to 4 = “Urge occurs 75%-100% of the time you do the tic”) and urge intensity on a 4-point Likert scale (1 = “minimal intensity/urge can be ignored for a considerable amount of time” to 4 = “strong intensity/urge needs relief almost immediately”). The clinician also asks about the bodily location of the urge. Three scores are generated: total number of distinct urges (I-PUTS Urge Number), total urge frequency (I-PUTS Frequency), and total urge intensity (I-PUTS Intensity).	Studies that have examined the psychometric properties of the I-PUTS have only done so in youth samples.
“Live urge monitor”	Brandt et al. (2016) (35)	Continuous, real-time self-rating of urge intensity	The urge monitor utilizes a slider controlled by a computer mouse to allow individuals to continuously rate their urge intensity in the present moment on a visual scale from 0-100, with ratings of the previous seconds displayed on the screen.	Mean intensity ratings have been shown to significantly correlate with 9-item PUTS scores (35–37)
“Urge thermometer”	Himle et al. (2007) (38)	Discrete, momentary self-rating of urge intensity	The scale captures a momentary assessment of the overall urge experience in a 0-8 or 0-9 range, where quantitative values are depicted on ascending bars with corresponding qualitative descriptions (e.g., “0” = “not at all” to “9” = “very, very much”). The urge intensity rating is prompted by a computer.	

*The University of São Paulo Sensory Phenomena Scale (USP-SPS) assesses a broader range of sensory phenomena occurring before repetitive behaviors, including, but not limited, to tics (61). Because the scale is not specific for the measurement of premonitory urges in tic disorders, it was not included in the review.

#### 3.1.1 Premonitory urge for tics scale

The most commonly used questionnaire was the Premonitory Urge for Tics Scale (PUTS), a 10-item, self-report scale developed by Woods et al. (12). The questionnaire contains 9 statements about premonitory urges and 1 statement about tic suppressibility. Respondents indicate their degree of agreement with each statement on a 4-point Likert scale ranging from 1 (“Not at all”) to 4 (“Very much”). Regarding content validity [i.e., the extent to which the phenomenon of interest is thoroughly represented by the questionnaire items (13)], a description of the two-stage method for PUTS item selection is provided in Woods et al’s original publication. In the initial validation study, PUTS item 10 (“I am able to stop my tics, even if only for a short period of time”) correlated poorly with the rest of the scale (12), a finding subsequently replicated in multiple other studies (14–19). Consequently, the tenth item is often excluded from the PUTS total score. **Supplementary Table S1** contains a full summary of publications examining psychometric properties of the PUTS.

The original PUTS, developed in English, has been translated into multiple languages: German, Greek, Hebrew, Italian, Japanese, Korean, Mandarin/Cantonese, and Spanish. The translation process is described in some (20–26) but not all (14, 15, 17, 27) studies. No studies involving non-English versions of the PUTS reported re-assessing content validity of the translated scale, though this scoping review did not encompass non-English publications.

Twenty studies (12, 14–32) examined internal consistency of the PUTS, with Cronbach’s alpha values for the 9-item PUTS ranging from 0.75 (23, 27, 31) to 0.85 (24) in sample populations ranging in size from 22 (15) to 656 (17). Seven of these studies assessed internal consistency across children of different age groups within their sample, typically stratifying participants into those 10-years-old and younger and those older than 10 years (12, 17, 18, 20–23). Four of these studies found lower Cronbach’s alpha values (ranging from 0.57 to 0.70) in the younger age group (12, 18, 20, 21), but the other three studies did not observe this pattern (17, 22, 23). Three studies observed similar Cronbach’s alpha values between youth (< 18- years-old) and adults (14, 15, 24). Overall, the 9-item PUTS has shown acceptable internal consistency, with conflicting findings on the scale’s internal consistency in tic disorder populations younger than 10 years of age.

PUTS scores have been found to be temporally stable over a range of less than two weeks to four months (12, 19, 22, 23, 26), with between-timepoint correlations ranging from 0.49 (22) to 0.89 (23). In the largest of these studies, Li et al. observed a between-timepoint correlation of 0.89 at one month in 147 children with tic disorder (23), and Reese et al. observed a between-timepoint correlation of 0.79 at two weeks in 122 adolescents and adults with CTD (19). A single study examined differences in temporal stability across age groups, administering the Spanish version of the 9-item PUTS at baseline and 4-month follow-up (22). The correlation between scores was significant and positive for the subgroup older than 10 years of age and non-significant for the subgroup 10-years-old and younger (sample sizes of these subgroups were not reported). Notably, to assess temporal reliability of the PUTS, the above studies either employed Pearson’s correlations or did not report the specific correlation method used (12, 19, 23); no study reported intraclass correlations.

A number of studies conducted factor analyses to assess the dimensions of premonitory urge captured by the PUTS, with mixed results (14, 15, 17, 18, 22–24, 26). Study sample sizes varied widely, ranging from 38 (26) to 656 (17) participants, with many studies involving fewer than 100 participants (15, 18, 22, 24, 26). Multiple studies found that items 4 and 5 loaded onto a factor interpreted as “OCD-related items” or “just right phenomena” (17, 22, 24, 26). Other commonly proposed factors included “quality” (14, 15, 18, 22, 24, 26) and “intensity” (14, 15, 23, 24), although the items composing these factors were inconsistent across studies.

A single study, in a sample of 102 adults with TS, examined floor and ceiling effects of the PUTS (16). Both floor (3.9%) and ceiling (2.0%) effects were found to be small.

#### 3.1.2 Individualized premonitory urge for tics scale

Whereas the PUTS is a self-report questionnaire, the Individualized Premonitory Urge for Tics Scale (I-PUTS) is a clinician-administered scale. The clinician assesses the presence, bodily location, frequency, and intensity of urges for individual tics experienced over the past week, employing a checklist analogous to the tic checklist in the Yale Global Tic Severity Scale (YGTSS). Each urge is rated on a 4-point Likert scale for both frequency and intensity. The scale generates three scores: urge number, urge frequency, and urge intensity (33). The authors note that the I-PUTS, in contrast to the PUTS, permits assessment of individual premonitory urges, quantifies premonitory urge in three separate dimensions, and specifies the timeframe in question.

In the initial validation study, McGuire et al. administered the I-PUTS to 75 children with tic disorders and found excellent inter-rater reliability (33). The I-PUTS scores did not significantly correlate with scores from scales assessing severity of common comorbid psychiatric symptoms. In contrast, the PUTS score, in the same study, did significantly correlate with some psychiatric symptom severity scores, suggesting that the I-PUTS possesses better divergent validity than the PUTS. The three I-PUTS scores showed small correlations (I-PUTS number: r = 0.28, p < 0.02, I-PUTS frequency: r = 0.23, p < 0.05, I-PUTS intensity: r = 0.19, p = 0.10) with the 9-item PUTS score. The study did not examine the test-retest reliability of the I-PUTS. Che et al. (34) translated the I-PUTS into Chinese, describing their methods for doing so, and then administered the resultant Chinese I-PUTS to 123 children with tic disorder. They found good internal consistency, both for children 8-10-years-old and children 11-14-years-old; good test-retest reliability at one month; and good inter-rater reliability for all three I-PUTS scores (34). The three Chinese I-PUTS scores exhibited significant, medium-sized positive correlations with the Chinese PUTS score (I-PUTS number: r = 0.433, p < 0.01, I-PUTS frequency: r = 0.486, p < 0.01, I-PUTS intensity: r = 0.489, p < 0.01).

#### 3.1.3 Urge monitors

In contrast to the above studies employing questionnaires or structured interviews, several studies have used a “live urge monitor” (15, 35–37) or an urge thermometer (38–42) to precisely gauge premonitory urge intensity over short time periods. In a factor analysis including real-time urge intensity (average live urge monitor score over five minutes) and the 10 PUTS items, real-time urge intensity loaded on the second of the factors, which the authors interpreted as “intensity” of premonitory urges (15).

### 3.2 Characteristics of premonitory urge

#### 3.2.1 Prevalence of premonitory urges

Prevalence estimates of premonitory urges in individuals with tic disorders range from 37% (43) to 93% (3, 19, 34, 43–51). Prevalence rates vary by age of the sample population. In a sample of 656 children with CTDs, prevalence of premonitory urges, as assessed by the PUTS, was 81% for children 7-years-old and younger, 95.5% for children 8-10-years-old, and 97.5% for children 10-years-old and older (17). The following two studies, which asked individuals categorical questions about the presence of premonitory urges (“yes/no” or “yes/no/don’t know” response options) rather than administering a scale, observed lower prevalence estimates. In Sambrani et al’s study of 1,032 individuals with tic disorders, premonitory urges were reported by 46.7% of those less than 10-years-old, 61.3% of those 10-12-years-old, and 79.7% of those 12-years-old and older (50). In Banaschewski et al’s study of 251 children and adolescents with TS, premonitory urges were reported by 24% of 8-10-year-olds, 34% of 11-14-year-olds, and 57% of 15-19-year-olds (43).

#### 3.2.2 The nature and subjective quality of premonitory urges

In a sample of 122 adolescents and adults with CTDs, the most frequently endorsed premonitory urge qualities from the PUTS were “an energy in my body that needs to get out” (>80%) and “an inner feeling of being wound up or tense” (>80%) (19). In another study of 656 youth with CTDs, “an energy in my body that needs to get out” was again the most frequently endorsed urge quality from the PUTS (17). Schunke et al. (52) administered a questionnaire to 14 adults with TS, allowing participants to select the most accurate descriptors for their premonitory urges from a list of 14 different qualities. The three most selected qualities were inner urge (100%), increased tension (71%), and urge to move (64%) (52). In a sample of 291 adults with CTDs, the following premonitory urge qualities were endorsed, in order of decreasing frequency (based on the publication’s Supplementary Table S1): “feelings of tension” (61%), “pressure” (45%), “feeling discomfort” (43%), “a feeling of energy that needs to be released” (40%), “a not-just-right feeling” (33%), “the feeling that something was building up” (31%), “an itch” (29%), and “incompleteness” (21%) (53). Participants also rated urge intensity on a 1-to-11-point scale in this study; urge intensity did not significantly differ between the above urge qualities. In contrast to the more quantitative approaches discussed above, two case reports and series provide narratives of individuals with tics describing their experience of premonitory urges (3, 54).

The quality of premonitory urges appears to differ in tic disorder subgroups with obsessive-compulsive disorder (OCD) and/or attention-deficit/hyperactivity disorder (ADHD) compared to those without these comorbidities. In particular, multiple studies have observed increased prevalence (47, 53) and/or intensity (19) of the “not-just-right” quality of premonitory urges in individuals with tic disorder and comorbid OCD or obsessive-compulsive symptoms. In a study of 74 individuals with CTDs, Edwards et al. found that the subgroup of participants with comorbid ADHD and/or OCD reported greater “tense” or “wound up” feelings (PUTS item 3) than those without comorbid ADHD or OCD, though PUTS total scores did not significantly differ between groups (32).

Based on only two small studies, premonitory urges are distressing for some individuals with tics. In Cohen and Leckman’s study, 57% of 28 participants reported their urges to be “more bothersome than the tics themselves” (3). In more recent interviews of 42 youth with TS, some found their urges to be “uncomfortable or painful” and perceived reducing their urges as “an important outcome of treatment” (55).

#### 3.2.3 Number and frequency of premonitory urges

Among individuals who experience premonitory urges, significant variability exists as to the number and frequency of the urges. In one study with 135 adults and children with tic disorders, participants endorsed an average of 8.7 distinct urges (48), while in a different study of 75 children with tic disorders, participants endorsed an average of 2.9 distinct urges (33). Among a sample of 50 children and adults with TS, 92% reported premonitory urges, but only 35% endorsed premonitory urges with all of their tics (47). In this same study, only 3% of participants reported urges with fewer than 25% of their tics. In a sample of 75 children with tic disorders, McGuire et al. found that for a given tic with an associated premonitory urge, participants “experienced urges over 50% of the time they had the tic” (33).

#### 3.2.4 Premonitory urge location

In their seminal study, Cohen and Leckman found that, of 28 children and adults with TS, 45% felt premonitory urges “in their mind,” while the other 55% felt the urge “in another body area” (3). In a subsequent study of children and adults with tic disorders, Leckman et al. found that, of 123 participants with premonitory urges, 89% reported their urges were “either partly or wholly a physical experience” as opposed to a mental one (48). O’Connor et al. posed a similar question to 60 adults with CTDs and found that, of the participants that experienced premonitory urges (>80%), only two reported the sensation was mental as opposed to physical (49). In their aforementioned study, Leckman et al. found that 40% of participants felt the urge exclusively in muscle, 24% felt it in both muscle and joints, and 8% felt it exclusively in joints (48). Similarly, Kwak et al. found that urges were more frequently perceived to occur in muscles than in joints or skin (47). Among 291 adults with CTDs, premonitory urges for motor tics were most frequently felt “exactly at the location or in direct proximity to the corresponding tic” (44). However, 52.6% of participants were unable to report a location for at least one premonitory urge (“diffuse, non-circumscribed premonitory urge”), and 3.9% of participants endorsed an urge located “outside the body” for at least one of their tics. In this study, a diffuse premonitory urge location was more common for complex tics than simple tics and more common for vocal tics than motor tics (44). In a large study of 700 children and adults with tic disorders who experienced premonitory urges, 64% reported their urges were localized, 16% reported their urges were diffuse, and 13.7% were unable to identify the precise nature of their urges (50). These findings generally align with those of a smaller study of 19 children and adults with TS: 53% reported their urges as focal sensations, 11% as generalized sensations, and 37% as both generalized and focal sensations (56).

The face and shoulders have tended to predominate as the most common bodily locations for premonitory urges (33, 34, 44, 47, 48, 52). Two studies utilized the I-PUTS to determine the relative intensity of premonitory urges across bodily locations. In these studies, urges in the “whole body/other region” (33) and the “neck/throat region” (34), respectively, were rated as most intense.

#### 3.2.5 Development of premonitory urges over time

As aforementioned, prevalence rates of premonitory urge increase over the course of development (17, 43, 50). The trajectory of premonitory urges, however, is unclear due to a paucity of longitudinal investigations. In their cross-sectional study of 135 children and adults with tic disorders, Leckman et al. found that individuals who experienced premonitory urges (n = 123) recalled first becoming aware of their urges at 10.0-years-old (SD = 6.2 years) (48). Awareness of premonitory urges emerged an average of 3.1 years (SD = 5.7 years) after tic-onset. Gulisano et al. administered the PUTS to 95 children with TS at baseline (mean age = 7.3 years, SD = 1.5 years) and at long-term follow-up (mean age = 13.1 years, SD = 3.7 years), observing that mean PUTS score was significantly higher at follow-up (24.1) than at baseline (13.5) (21).

Multiple cross-sectional studies have compared measures of premonitory urge severity between different age groups. Kyriazi et al. found that children with tic disorder who were 12-15-years-old (n = 13) had significantly higher PUTS scores than those who were 6-11-years-old (n = 39) (25). Yet, other studies have failed to identify significant differences in PUTS scores between older and younger youth (12, 18, 20, 23) or between adults and youth (24, 26). Langelage et al. (37) collected data using a live urge monitor over five minutes in 25 youth (8-18-years-old) with TS and compared their results to those from Schubert et al. (36), a similar study in 21 adults with TS (mean age = 30.5 years, SD = 10.6 years). They found that average urge monitor intensity ratings during the task did not significantly differ between youth and adult samples.

Other cross-sectional studies have examined the correlation between measures of premonitory urge severity and age. In some studies of children and adolescents, age positively correlated with premonitory urge severity, as indexed by the PUTS (29, 33), but other studies failed to identify a significant correlation using the PUTS (34, 57, 58) or the I-PUTS (33, 34). Most studies involving adults have not observed a significant correlation between age and PUTS score (16, 19, 59, 60), though one small study of 18 adults and children with TS did observe a significant positive association (61). In contrast, Brandt et al., assessing urge intensity on an 11-point Likert scale in 291 adults with CTDs, found that younger age was associated with more intense urges; however, older age was associated with a greater likelihood of experiencing urges (dichotomous variable), after accounting for gender and psychiatric comorbidity (53).

#### 3.2.6 Premonitory urge relationship to tics

Likelihood of premonitory urges appears to vary by tic location, complexity, and severity. Tics of the head, neck, and shoulders were most commonly associated with a premonitory urge in a sample of 135 children and adults with tic disorders (48). In another study of 291 adults with CTD, premonitory urges were reported for 80% of complex motor tics compared to only 67% of simple motor tics (44). Among 240 individuals with CTDs, tics with an associated premonitory urge were on average rated as more severe compared to tics without an associated urge (62).

Evidence indicates that tics generally alleviate premonitory urges. Reese et al. found that 85% of 122 older adolescents and adults with CTD endorsed PUTS item 9 (“After I do a tic, [the sensation] goes away at least for a little while”) (19), and Essing et al. (44) found that 97% of 291 adults with CTD reported relief from their urges following at least one of their tics. In two studies involving both children and adults with TS (with sample sizes of 28 and 50), over three-fourths of participants (75% and 83%, respectively) reported relief from premonitory urge following a tic (3, 47). Moreover, 71% (3) and 67% (47) of participants believed their tics were voluntary responses to their premonitory urges. In the latter of these two studies, 88% of participants endorsed an increase in urge intensity if prevented from executing their motor tics (47).

A few studies have utilized a live urge monitor to examine the relationship between urges and tics in real time. Brandt et al. assessed real-time urge intensity in 16 adults with TS using a live urge monitor while two independent raters recorded tic occurrence (35). In both free ticcing and tic suppression conditions, urge intensity tended to increase prior to a tic and decrease after a tic. In a separate study of 22 adults with TS, number of tics during a five-minute live urge monitor task positively correlated with average real-time urge intensity (15). Two similar studies (36, 37) found that greater real-time urge intensity was associated with increased likelihood of real-time tic occurrence and tic intensity. In a study of 11 adolescents and adults with CTDs, Wellen et al. used a live urge monitor and video-based tic rating to assess urges and tics (63). They observed that real-time urge intensity was positively associated with tic occurrence in the subsequent second for nine (82%) of the participants. Additionally, a relationship between tic occurrence in the previous second and urge intensity in the subsequent second was significant for eight (73%) of the participants, but for seven of these eight participants the relationship was such that tic occurrence predicted an increase in urge intensity in the subsequent second. Six of these seven participants showed an eventual decrease of urge intensity following tics but at varying timepoints (four to nine seconds post-tic).

#### 3.2.7 Associations between premonitory urge severity and tic severity

Results are mixed concerning the cross-sectional association between premonitory urge severity and tic severity. Correlations of PUTS score with Yale Global Tic Severity Scale-Total Tic Score (YGTSS-TTS) and YGTSS Impairment Score range widely, from 0.08 (20) to 0.63 (64) and from 0.02 (58) to 0.60 (65), respectively. In studies with sample sizes of more than 100 participants, YGTSS-TTS consistently correlated with PUTS score, with correlation values of 0.13-0.27 in youth samples (23, 29, 46) and 0.20-0.32 in adult samples (19, 66). **Supplementary Table S2** contains detailed results from studies examining the relationship between PUTS score and measures of tic severity. The relationship between I-PUTS scores and tic severity has been less extensively studied, but the existing data demonstrate a positive relationship between I-PUTS and YGTSS scores [(33, 34, 67, 68); **Supplementary Table S3**].

In the sole study to examine the relationship between premonitory urge severity and long-term tic severity, 80 CTD participants aged 16-30 years completed the PUTS and YGTSS at baseline and at a long-term follow-up (mean = 11.2 years later) (69). Baseline PUTS score did not significantly predict follow-up YGTSS-TTS or Impairment Score after controlling for selected covariates, nor did baseline PUTS score predict change in tic impairment over time. However, baseline PUTS score did significantly predict change in YGTSS-TTS between baseline and follow-up. That is, those with more severe premonitory urges at baseline experienced less reduction in tic severity over time.

#### 3.2.8 Associations between premonitory urge severity and tic suppression ability

Several studies have evaluated the relationship between self-reported tic suppression ability and premonitory urge severity. Cohen and Leckman (3) found that 55% of their 28 participants felt their urges enabled them to better suppress their tics, but in Leckman et al.’s subsequent study (n = 132, age range 8-71-years-old, with 123 participants endorsing premonitory urge), only 20% of participants reported their urges enhanced their tic suppression ability (48). Among 1,032 children and adults with any tic disorder, presence of premonitory urge was positively associated with reported ability to suppress tics (50). Conversely, in a sample of 62 children and adults with TS, Matsuda et al. found no significant correlation between PUTS score and the tic suppression ability sub-score of the Tic Suppression Scale (24). However, in the subgroup of participants without comorbid OCD or ADHD (n = 37), these two scores showed a significant negative correlation, such that those with greater premonitory urge severity reported less ability to suppress tics. Within the 8-10-year-old subgroup (n = 81) of a sample of 254 youth with TS, those who endorsed premonitory urges were 2.75 times more likely to report tic suppressibility compared to 8-10-year-olds who did not endorse urges (43). In the other age groups, those who endorsed premonitory urges were not significantly more likely to report the ability to suppress tics.

Three studies have evaluated the relationship between objectively measured tic suppression ability and premonitory urge severity. In two studies of adults with TS, PUTS score did not significantly correlate with tic inhibition potency, as assessed by percent difference in the Modified Rush Video-Based Tic Rating Scale (MRVS) score during free-to-tic and tic inhibition conditions (70, 71). In their study of children with CTDs, Capriotti et al. included a condition in which participants were instructed to suppress tics but had the option to initiate a 10-second break during which they were free to tic (40). After removing one outlier, PUTS score positively correlated with average number of breaks taken.

#### 3.2.9 Psychosocial impact of premonitory urge

Two cross-sectional studies have examined the association between premonitory urge severity and the impact or consequences of ticcing. Zinner et al. administered a neuropsychiatric battery, including the PUTS, and a questionnaire assessing peer victimization and bullying behavior to 211 parent-child dyads, in which all children had a CTD (28). Based on questionnaire results, children were classified as “victims,” “bullies,” “bully-victims,” or “non-victims.” Severity of premonitory urge, as well as of tics, explosive outbursts, and internalizing symptoms, was greater in “victims” compared to “non-victims.” The authors suggested that peer victimization perceived to be tic-related may increase the aversive experience of tics, which in turn may intensify premonitory urges. Consistent with the above study results, Capriotti et al. observed in their questionnaire-based study of 118 youth with TS that tic-related impact score predicted PUTS score after adjusting for tic severity and comorbid psychiatric diagnoses (72).

### 3.3 Associations between premonitory urges and other clinical characteristics

#### 3.3.1 Interoception

Three studies have examined the potential association between premonitory urge severity and interoceptive sensibility, defined as “self-reported sensitivity to bodily sensations” (73), with differing results. In a sample of 18 adolescents and adults with TS, PUTS score and Private Body Consciousness scale score did not correlate (74). In contrast, in 21 adults with TS, score on the “awareness” section of the Body Perception Questionnaire significantly positively correlated with PUTS score (73), and in 48 adults with CTDs, a Multidimensional Assessment of Interoceptive Awareness, Version 2 (MAIA-2) composite score was significantly associated with PUTS score after adjusting for tic and OCD symptom severity (75), indicating that participants with greater interoceptive sensibility reported more severe premonitory urges.

Several studies have examined the potential association between premonitory urge severity and interoceptive accuracy and/or interoceptive awareness. Interoceptive accuracy is a construct that reflects “objective interoceptive performance” (73) on tasks such as the heartbeat counting task, whereas interoceptive awareness is a construct defined as “insight into interoceptive ability, reflecting correspondence between subjective and objective measures” (73). In two studies assessing interoceptive accuracy in youth with CTDs (respective tic disorder sample sizes of 28 and 29), accuracy on a heartbeat perception task did not correlate with PUTS score (27, 30). One of these studies also assessed interoceptive accuracy (IAcc) by examining the correlation between self-rated muscle tension in the corrugator and masseter muscles and electromyography (EMG) activity, finding that PUTS score significantly positively correlated with Masseter IAcc but not Corrugator IAcc (27). In a study of 21 adults with TS, PUTS score did not correlate with participants’ accuracy in a heartbeat perception task or a heartbeat discrimination task nor did PUTS score correlate with metacognitive insight into accuracy on those two tasks (interoceptive awareness) (73). In contrast, in 19 adults with TS, better performance on a heartbeat counting task was significantly associated with higher PUTS score after adjusting for tic and OCD symptom severity (76).

A single study has examined the relationship between premonitory urge and embodiment. Rae et al. recruited 23 adults with TS to complete the rubber hand illusion task (77). PUTS score was not significantly associated with “body ownership plasticity” as indexed by the proprioceptive drift between synchronous and asynchronous conditions. However, PUTS score was correlated with embodiment prediction error, a metric generated from the difference between proprioceptive drift (objective measure of embodiment) and self-ratings of rubber hand embodiment during synchronous stimulation (subjective measure of embodiment). In this study, PUTS score was also associated with change in the “experience of rubber hand ownership” between synchronous and asynchronous conditions.

#### 3.3.2 Exteroception

Several studies have examined associations between premonitory urges and self-reported perception of the external environment, again with mixed results. In a study of 18 children and adults with TS, PUTS score did not significantly correlate with scores from the Sensory Gating Inventory or the Structured Interview for Assessing Perceptual Anomalies (61). In contrast, in a sample of 34 adults with CTDs, PUTS score significantly correlated with both Sensory Gating Inventory and Sensory Perception Quotient scores, indicating that participants with greater premonitory urge severity reported more sensory hypersensitivity, defined as “heightened awareness of and reactivity to external stimuli” (31). In a sample of 140 children and adults with TS, presence of premonitory urges was not significantly associated with presence of stimulus sensitization, defined as “heightened sensitivity to tactile, auditory, and visual stimuli that [result] in uncomfortable sensation, tension, or non-tic movement” (78). Whereas the above studies relied on self-reported sensory perception, Schunke et al. used the “Quantitative Sensory Testing” (QST) battery to objectively assess 13 sensory parameters, including thermal, mechanical, and pain thresholds, in 14 adults with TS (52). PUTS score did not significantly correlate with any of the sensory parameters (52).

#### 3.3.3 Cognitive traits and functions

Several studies have examined the associations between premonitory urges and various aspects of cognition and metacognition. In a study of 122 adolescents and adults with CTD, PUTS score showed a small but significant positive correlation with intelligence quotient (IQ) as measured by the Wechsler Test of Adult Reading (19). Two other studies, with samples of 42 (12) and 29 (57) children and adolescents, found no such association.

Sections 3.2.8 and 3.4.1 review investigations of premonitory urge and tic suppression ability, but two studies have examined the relationship of premonitory urges with facets of inhibition outside the context of tic suppression. In a sample of 18 adolescents and adults with TS administered the standard and emotional Stroop tasks, PUTS score negatively correlated with standard Stroop time difference but not with standard Stroop error difference or emotional Stroop time difference or error difference (74). The authors suggested that the negative correlation between standard Stroop time difference and PUTS score may indicate reduced inhibitory interference in those with more severe premonitory urges. In a study of 40 adults with TS, average number of escape blinks during a blink suppression task, which included emotional and neutral facial expression conditions, was not associated with PUTS score (79).

Ganos et al. assessed the experience of intention during voluntary action in 27 adolescents with TS (80). Shorter time interval between the experience of intention and action performance was associated with higher PUTS score. The authors suggested that severe premonitory urges may interfere with self-perceived volition, as urges create perceptual noise from which signals of volitional action must be discriminated.

Arbuzova et al. conducted an assessment of metacognitive ability in 21 adults with TS, using a forced discrimination task of tactile stimuli (first-order) and confidence ratings regarding the accuracy of participants’ judgements in this task (second-order) to calculate the M-ratio, reflecting “the sensitivity of subjective second-order reports relative to objective first-order task performance” (81). They found no significant correlation between PUTS score and M-ratio. In a sample of 23 adults with TS, PUTS score significantly correlated with score from the Thinking About Tics questionnaire (“tic-related cognitions”) but not from the Meta-Cognitions Questionnaire (41).

#### 3.3.4 Comorbid psychiatric symptoms/diagnoses, quality of life, and global functioning

Many studies, involving children, adolescents, and/or adults with tic disorders, have observed significant associations between severity of premonitory urges and OCD symptoms [(12, 15, 17, 19–21, 23, 24, 26, 29, 32, 50, 53, 59, 61, 65, 66, 75, 76, 82, 83); **Supplementary Table S5**]. Results are more ambiguous from studies that have examined associations between severity of premonitory urges and severity of ADHD symptoms, depression, and/or anxiety [(12, 15, 17, 19–21, 26, 29, 33, 37, 53, 59, 75, 82–85); **Supplementary Tables S6**, **S7**]. A smaller number of studies have examined the association between premonitory urges and various behavioral, conduct, and emotional problems, including distress tolerance and externalizing behavior [(12, 17, 21, 33, 51, 58, 60, 86); **Supplementary Table S8**]. Of the studies that have assessed the relationship between premonitory urges and quality of life or global functioning, most indicate that more severe premonitory urges are associated with greater disability, poorer global functioning, and poorer health-related quality of life [(37, 53, 58, 59, 65, 66, 71, 72, 75, 82, 83, 87, 88); **Supplementary Table S9**].

#### 3.3.5 Other characteristics

Of the eight studies that have examined differences in PUTS or I-PUTS scores between males and females, no between-sex differences were evident (16, 19, 29, 32, 34, 59–61). However, in a sample of 1,032 children and adults with any tic disorder (mean age = 20.9 years, SD = 12.9 years; male-female proportion of sample not reported), males were more likely to report presence of premonitory urges than females (males: 72.3%, females: 65.1%) (50). Conversely, in a sample of 291 adults with CTDs (mean age not reported but 33.3% of sample older than 35 years of age; 24% female), females were more likely to report premonitory urges than males, after adjusting for age and comorbid diagnoses (53). In their study of 74 youth and young adults with CTD, Edwards et al. found that the relationship between PUTS score and self-reported diagnoses of ADHD and OCD did not differ by sex (32).

In a study of 60 individuals (mean age = 32.2 years, SD = 14.1 years) with TS, Eddy and Cavanna administered the PUTS and assessed coprolalia and mental coprolalia (urge to swear as a tic) using a non-obscene socially inappropriate symptoms (NOSIS) questionnaire. PUTS score did not significantly correlate with the presence of coprolalia or mental coprolalia (89). In a separate study of 60 individuals (mean age 33 years, SD 14 years) with TS, Eddy and Cavanna found that those who reported NOSIS (n = 40) had significantly higher PUTS scores than those who did not (n = 20) (90).

A study of 1,032 children and adults with any tic disorder observed a significant association between the presence of premonitory urges and “not just right experiences” (50). In 111 adults with TS, scores from the Non-Just-Right-Experiences Questionnaire (NJRE-QR) and the Feelings of Incompleteness Questionnaire significantly correlated with PUTS score (66). Notably, PUTS item 4 (“Right before I do a tic I feel like something is not ‘just right’ “) score only moderately correlated with NJRE-QR score, suggesting that PUTS item 4 and the NJRE-QR may not index the exact same construct (66).

### 3.4 Premonitory urge response to intervention

#### 3.4.1 Response to tic suppression

Mixed results have emerged from studies examining the effect of tic suppression on premonitory urge. Several have identified increased urge intensity during tic suppression. In a study of 13 children with CTDs, participants engaged in a tic suppression condition during which they had the option to initiate a 10-second break to tic freely (40). Participants rated their urge intensity on an “urge thermometer” at the initiation and termination of each break. Across participants, urge intensity significantly decreased between break initiation and termination. In this same study, the researchers also observed that average urge ratings were significantly higher in a reinforced tic suppression condition compared to the free-to-tic baseline condition. In a study of 45 children and adolescents with TS, participants completed a free-to-tic condition, a tic suppression condition, and an urge-acceptance condition, in which they were instructed to monitor “urges while practicing willingness to experience the urge and giving up any fight” (91). During each of these two-minute conditions, participants rated the intensity of each urge that occurred on a 1-9-point scale, allowing for assessment of both urge intensity and frequency. Urge intensity, but not frequency, was significantly higher during the tic suppression condition compared to the free-to-tic condition. Interestingly, both urge intensity and frequency were significantly lower in the urge acceptance condition compared to the free-to-tic condition. Brabson et al. conducted a study in which 12 children with CTD completed alternating baseline and differential reinforcement of zero rate behavior (DRO; i.e., incentivized tic suppression) conditions (42). Two participants showed an “M” pattern, with urge intensity lower in baseline conditions and higher in DRO conditions; three participants showed a “W” pattern, with urge intensity higher in baseline conditions and lower in DRO conditions; and the remaining participants showed other, less clear patterns of urge intensity across conditions. In a study of 16 adults with TS, participants rated their premonitory urge intensity on a live urge monitor during tic suppression and free-to-tic conditions (35). The timing of peak urge intensity differed between conditions: in the free-to-tic condition urge intensity peaked just after tic occurrence, while in the tic suppression condition urge intensity peaked just before tic occurrence. Notably, urge intensity tended to increase over the course of the suppression condition.

In contrast to the above, several studies have observed no change in premonitory urge intensity during tic suppression. In a study of 12 children and adolescents with CTD, participants completed three 10-minute free-to-tic conditions and two 40-minute DRO (incentivized tic suppression) conditions (39); urge intensity was rated every 10 seconds on an “urge thermometer.” Aggregate urge intensity did not significantly differ between baseline and DRO conditions, but tic frequency was significantly lower in DRO conditions. In a study of 11 individuals 17-60-years-old with CTD, participants engaged in multiple behavioral conditions, including free-to-tic (10 minutes each, 3 separate occurrences, though only first occurrence was used in analyses), tic suppression (30 minutes, 1 occurrence), and habit reversal tic suppression (tic suppression with a competing response; 30 minutes, 1 occurrence), while rating their urge intensity with a live urge monitor (63). Most participants showed similar urge levels across conditions, even though tic frequency was lower in the tic suppression and habit reversal tic suppression conditions compared to the free-to-tic condition. In a study of 22 adults with TS, participants completed the PUTS at baseline, suppressed their tics for 10 minutes, and then completed the PUTS again just before the end of the 10-minute task (70). The PUTS was also administered at 10 minutes, 20 minutes and 30 minutes post-suppression task. Mean PUTS score did not significantly differ between any of the timepoints. In study of 23 adults with TS, Hermann et al. assessed urge intensity in five conditions during which participants were instructed to focus their attention on different objects or phenomena: a picture of an empty room (baseline), a live video of themselves, a video of themselves ticcing, various objects within a room (distraction condition), and uncomfortable aspects of their tics (41). Participants completed each condition twice: once in a free-to-tic state and once in a tic suppression state. Attention condition, but not tic state, was significantly associated with urge intensity ratings. Relative to baseline, urge intensity was higher during the live feedback condition and the thoughts-about-uncomfortable-aspects-of-tics condition.

#### 3.4.2 Response to behavioral interventions

Nine studies have assessed whether various behavioral interventions, such as Comprehensive Behavioral Intervention for Tics (CBIT), exposure and response prevention (ERP), and habit reversal training (HRT), reduce premonitory urge severity (56, 92–99). **Supplementary Table S10** provides detailed results of these studies. Most studies employed the PUTS and observed no effect of behavioral interventions on PUTS scores. In a randomized controlled trial comparing 10 sessions of CBIT versus psychoeducation and supportive therapy (PST) in 122 adults with CTD, PUTS scores did not significantly differ between treatment groups, though PUTS scores did significantly decrease over time across both groups (93). Interestingly, in the companion pediatric study of CBIT versus PST (n = 126), premonitory urge severity did not significantly change over time for either treatment group (93). Notably, one study, investigating the effectiveness of ERP in 18 children and adults with TS, indexed premonitory urge severity with subjective units of distress rather than the PUTS: subjective distress from premonitory urges decreased both within and across 10 ERP sessions (56).

#### 3.4.3 Response to pharmacotherapy

Nineteen studies have assessed responsiveness of premonitory urges to medication (47, 84, 100–116). **Supplementary Table S11** contains detailed results of these studies. In a randomized, double-blind, placebo-controlled trial of topiramate for children and adults with TS (20 individuals completed the study), premonitory urge severity (as measured by clinical global impression), as well as tic severity, improved in the topiramate arm (101). In contrast, premonitory urge severity did not improve during open-label treatment with aripiprazole (84) or ecopipam (100). Three studies examined the effect of cannabis on premonitory urges and observed some benefit (111–113), though each study had notable limitations: one study solely interviewed individuals about their experience using marijuana, another study enrolled a small sample (n = 18) of adults with TS for open-label cannabis treatment using varying doses and administration methods, and the only randomized controlled trial (cross-over design) enrolled a small sample (n = 12) of adults with TS for a single dose. Botulinum toxin injections improved premonitory urge severity in a number of case series (47, 103, 104, 106, 107) and in one randomized controlled trial (n=18) (105), though it is notable that none of these studies assessed premonitory urge with a validated, symptom-specific scale. In a combined pharmacotherapy-behavioral intervention, McGuire et al. randomized 20 youth with TS or chronic motor tic disorder to one session of HRT with either D-cycloserine 50 mg or placebo (taken one hour pre-HRT session). The D-cycloserine group exhibited significantly more improvement in I-PUTS frequency score, but not I-PUTS intensity score, than the placebo group (102).

#### 3.4.4 Response to neural stimulation

Most studies assessing response of premonitory urges to deep brain stimulation (DBS) have involved small case series and open-label studies, with the majority demonstrating some degree of improvement. Among 18 TS participants (age range: 17-47-years-old) with bilateral DBS in the centromedian–parafascicular (CM–Pfc) and ventralis oralis (Voa) nuclei of the thalamus, nine underwent blinded rating in “on” and “off” DBS conditions nine months post-implantation (117). In the “off” condition, a subset of participants (precise number not reported in the publication) experienced return of their premonitory urges. In two adults with refractory TS who underwent bilateral DBS in the CM-Pfc-ventral oral nuclei of the thalamus, PUTS scores improved after implantation, with sustained improvement two years post-implantation (118). In a case report of a 17-year-old female with TS who received bilateral globus pallidus internus (GPi) DBS, PUTS score reduced from 36 to 15 two months after surgery, with further reduction to 9 two years post-surgery (119). In contrast to the above studies, a nine-month, randomized, double-blind crossover trial comparing DBS of the centromedian-ventro-oral internus of the thalamus (CM-Voi), the posteroventral lateral globus pallidus internus (pvl GPi), and sham stimulation in nine adults with TS found no significant differences in PUTS or premonitory urge visual analogue scale scores across stimulation targets (120).

In the single pilot study to examine the impact of transcranial direct current stimulation (tDCS) on premonitory urge, Eapen et al. administered tDCS to the anterior supplementary motor area (SMA) in two adult males with TS over the course of six weeks (121). At three months post-treatment, PUTS scores had improved for both participants.

Median nerve stimulation (MNS) is a non-invasive intervention currently under study to reduce tic severity. Among 16 adolescents and adults with TS six, one-minute rounds of 10 Hz-MNS decreased live urge monitor ratings by 33% (not significant) during stimulation periods (122). In the same study, results of a stepwise multiple regression model found that higher PUTS score corresponded to greater reduction in tic intensity during MNS. The study also found that, during MNS, reduction in live urge monitor ratings was associated with reduction in tic frequency. Subsequently, Morera Maiquez et al. conducted a double blind, sham-controlled trial of home-administered MNS, obtaining complete data from 117 participants (123). Participants were randomized into one of three groups: active stimulation, sham stimulation, or treatment as usual. The treatment consisted of four weeks of a daily, 14-minute stimulation protocol. Reductions in premonitory urge severity, as assessed by the Premonitory Urges for Tic Disorders Scale-Revised (PUTS-R), did not significantly differ between groups.

Fu et al. conducted a sham-controlled trial of repetitive transcranial magnetic stimulation (rTMS) (0.5 Hz, intensity 90% of resting motor threshold) to the bilateral parietal cortices in 30 adolescents and adults with TS (124). Participants received three rounds of 400 pulses per side (10 minutes between rounds) for 10 consecutive days. Symptom rating scales were administered at baseline, at the end of treatment, one week after treatment, and one month after treatment. PUTS scores decreased significantly more in the rTMS group, with persistent improvements one-month post-treatment. A similar pattern emerged for YGTSS global scores (i.e., sum of Total Tic Score and Impairment Score), YGTSS motor tic scores, YGTSS vocal tic scores, and MRVS.

### 3.5 Neural correlates of premonitory urges

#### 3.5.1 Functional imaging

Numerous functional imaging studies have examined the neural activity associated with premonitory urges, but cross-study comparisons are challenging due to between-study differences in task, imaging, and statistical methodology. That said, findings generally suggest a relationship of premonitory urge with functional activity and/or connectivity in sensorimotor cortical and subcortical structures, particularly the insula and SMA. **Supplementary Table S12** provides detailed results, including effect sizes and p-values, from all studies investigating neural correlates of premonitory urge.

In a resting state functional magnetic resonance imaging (fMRI) study of 13 adults with TS, PUTS score was associated with functional connectivity of the right dorsal anterior insula (dAI) with the right SMA2 and left SMA1 (125) (Table 1 in the cited publication reports MNI coordinates for “SMA1” and “SMA2”). In a separate resting state fMRI study of 40 adults with TS, PUTS score was negatively associated with connectivity between the insula and inferior frontal gyrus (IFG), between the orbitofrontal cortex (OFC) and pre- and post-central gyri, and between the putamen and IFG (126). In contrast to the above resting state fMRI studies, Tinaz et al. found no significant associations between PUTS score and functional connectivity of sensorimotor cortex (SMC) with any brain regions in 16 adults with TS (127).

Two studies have examined the relationship between premonitory urge and fMRI changes during suppression tasks. In the study by Ganos et al., 14 adults with TS completed free-to-tic and tic suppression conditions during fMRI (128). Regional homogeneity (ReHo), a fMRI measure of local connectivity, in the left IFG was greater during the tic suppression condition than the free-to-tic condition. However, ReHo contrast value in the left IFG showed no correlation with PUTS score. In the study by Bhikram et al., 40 adults with TS underwent fMRI while completing a blink suppression task that included free-to-blink and blink suppression conditions, during which participants were shown either angry or neutral facial expressions (79). Contrasts in brain activity during free-to-blink and blink suppression conditions were not significantly associated with PUTS score. However, when contrasting angry facial expression conditions with neutral facial expression conditions, greater activity in the thalamus, hippocampus, mid-cingulate, caudate, and middle temporal gyrus was associated with greater urge severity as quantified by the PUTS.

Other studies have investigated the relationship of premonitory urge severity to fMRI activation under a variety of task conditions that did not explicitly involve tic suppression. In 25 adults with TS completing a theory of mind task, PUTS score was associated with fMRI activity in the right temporal-parietal junction, left amygdala, and right amygdala (129). In a follow-up study using the same sample population, participants completed a reading-the-eyes task in which they made judgments about either mental state or age based on an image of eyes (130). In both task conditions, greater activity in the bilateral temporal-parietal junctions was associated with higher PUTS score. In a study of eight adults with TS, participants completed a visuospatial priming task during fMRI scan, before and after 10 weeks of CBIT (94). Prior to CBIT, task-related activation in the superior temporal gyrus was negatively correlated with PUTS score. The change in task-related activation from pre- to post- CBIT did not correlate with baseline PUTS score or change in PUTS score. In a study of 23 adults with TS, participants completed a modified “Go/NoGo” fMRI task (131). Greater representational similarity in the caudate nucleus between “go” and “no go” conditions was positively correlated with PUTS score, but the significance of this association did not survive correction for multiple comparisons. With the same sample population and Go/NoGo/Choose task, Rae et al. found no correlations of PUTS score with task effects for Choose-Go (“volitional action”) or Choose-NoGo (“intentional inhibition”) conditions (132). However, during trials in which participants “chose-go,” pre-SMA functional connectivity with the following regions was positively associated with PUTS score: caudate nucleus, globus pallidus, and thalamus. In a study of 21 adults with TS, participants completed a face perception task during fMRI in which they viewed angry or neutral faces and were instructed to report whether the face was male or female (133). Contrasts in both the neutral face and angry face conditions (relative to baseline) were not significantly associated with PUTS score. However, during the face perception task, functional connectivity between the insula and the following regions was positively associated with PUTS score: SMA, posterior cingulate, precuneus, and fusiform gyrus/cerebellum.

Three studies have examined associations between premonitory urges and neurotransmitter or neurotransmitter metabolite concentrations in different brain regions. Tinaz et al. used GABA ^1^H magnetic resonance spectroscopy (MRS) to measure GABA concentration in the sensorimotor cortex (SMC) in 16 adults with TS: GABA+/Creatine ratio in the SMC did not significantly correlate with PUTS score (127). In a larger study of 68 children with TS, He et al. used proton-edited MRS to assess concentrations of GABA and glutamate + glutamine (Glx) in the right insula, right SMC, and right SMA (134). After correcting for multiple comparisons, SMA GABA+ concentration significantly negatively correlated with I-PUTS frequency, intensity, and number scores such that lower level of SMA GABA+ was associated with greater premonitory urge severity. There were no significant associations of I-PUTS scores with GABA+ concentration in the SMC or with Glx concentration in any of the three brain regions of interest. In those without co-occurring ADHD, GABA+ level in the insula was negatively associated with all three I-PUTS scores, but in those with co-occurring ADHD these associations were either nonexistent or positive. A separate MRS study in 37 adults with TS found a significant negative association between thalamic glutamate concentration and PUTS score (135).

Some studies have examined the neural correlates of premonitory urge without a formal assessment of urge, rather inferring the neural correlates of premonitory urges by examining brain activity prior to tics or instructing participants to imagine the experiences of urges and tics. Hampson et al. conducted a study of 16 adults with CTD and 16 matched controls without any tic disorder (136). During fMRI scan, CTD participants were free to tic, and controls were instructed to imitate the tics of the CTD participant to which they were matched. Based on data from 14 CTD participants with premonitory urges and their matched controls, SMA activity was greater before and after tics in CTD participants compared to before and after intentional movements in controls. In a study of 10 adults with TS undergoing fMRI during a free ticcing condition, the following regions had stronger fMRI activation two seconds prior to tic-onset than at tic-onset: bilateral insula, bilateral posterior putamen, bilateral anterior cingulate cortex, bilateral SMA, and bilateral parietal operculum (137). In a study with data from 23 individuals (mean age = 26.3 years, SD = 6.4 years) with TS, participants underwent fMRI during multiple conditions, including one in which they imagined a situation that promoted tic occurrence (“urge phase”) and a condition in which they imagined performing a tic (138). Following these conditions, participants rated their distress or relief on a 5-point scale. Distress rating during the urge phase was positively associated with activation in multiple cortical and subcortical areas (**Supplementary Table S12** provides further details).

#### 3.5.2 Structural imaging

Several studies have sought to identify structural correlates of premonitory urges, generally identifying associations between premonitory urge severity and structural alterations in the insula and sensorimotor cortical regions. In a combined sample of 28 children and adolescents (mean age = 14.6 years, SD = 3.4 years) with TS, PUTS score positively correlated with grey matter volume in clusters within the anterior-dorsal right insula (139) and the left hemisphere posterior mid-cingulate cortex (140). In contrast, Draper et al. found no positive correlations between PUTS score and cortical grey matter thickness in any brain region in 29 children and adolescents (mean age = 14 years, SD = 3.1 years) with TS (57). However, PUTS score was negatively correlated with grey matter thickness in clusters within the right Rolandic operculum, left inferior occipital gyrus, left insula, and left pre-central gyrus. In a study of 28 adolescents with TS, focused on regions of interest within the cerebellum, PUTS score was positively correlated with grey matter volume in a cluster within the right cerebellar lobule VI (141). In 15 adults (mean age = 30.9 years, SD = 9.9 years) with TS, Tinaz et al. found no significant correlations between PUTS score and sensorimotor cortical volume (127). In contrast, in a larger study of 34 adults (mean age = 32.4 years, SD = 11 years) with TS, PUTS score positively correlated with grey matter volume in the left somatosensory cortex, dorsal left premotor cortex, and right prefrontal cortex (PFC) and with cortical thickness in the left somatosensory cortex and right PFC (142). Also, PUTS score was negatively associated with fractional anisotropy of white matter for portions of the bilateral superior longitudinal fascicles. Sigurdsson et al. used diffusion tensor imaging to assess white matter microstructure in 35 adolescents with TS, finding no association between diffusion indices and PUTS score (143). However, in a regression analysis to predict graph theory metrics in 24 regions of interest from clinical scales, adjusting for age, IQ, and total intracranial volume, the authors identified significant positive associations between PUTS score and local efficiency (“efficacy of information transfer”) in the right posterior insula and left anterior insula, as well as a significant negative association between PUTS score and local efficiency in the left caudate.

#### 3.5.3 Neurophysiological approaches

Several studies have used neurophysiological methods, including electroencephalography (EEG), magnetoencephalography (MEG), and TMS, to study premonitory urge. Collectively, findings are mixed across these studies, which enrolled small sample populations and employed diverse methodologies.

Loo et al. recorded EEG activity during cued eyes blinks in 23 children with CTDs and examined the correlation of premonitory urges with spectral power in four cortical areas (144). Urge strength, as measured by a subset of PUTS items, positively correlated with alpha power in the left parietal cortex and with gamma power in the dorsolateral PFC and SMA. Urge strength negatively correlated with alpha power in the posterior cingulate cortex. Urge presence, as measured by a different subset of PUTS items, also positively correlated with gamma power in the SMA. In a sample of 11 adults with TS (PUTS data for n = 8), Niccolai et al. used MEG to assess beta power change in the motor and premotor cortices during time periods in which participants were free to tic (145). Two pre-tic time windows were defined: 1-0.5s pre-tic and 0.5-0s pre-tic. Correlations between PUTS score and beta power slope in each time window were not significant. However, beta power slope in the 1-0.5s pre-tic time window significantly correlated with PUTS item 6 (“I feel like there is energy in my body that needs to get out”) score, which the authors interpreted as reflective of motor urges. In 16 adults with TS, Tinaz et al. used MEG to measure (baseline) beta power in the sensorimotor cortex and found no significant correlation between this MEG measure and PUTS score (127).

Sigurdsson et al. used neuro-navigated transcranial magnetic stimulation (nTMS) in 16 children and adults with TS to map the representation of different muscles in the sensorimotor cortex (146). PUTS score positively correlated with the area of first dorsal interosseous (FDI) cortical representation. Additionally, PUTS score negatively correlated with the Euclidean distance between the FDI representation site and the representation sites for the orbicularis oris, masseter, and orbicularis oculi. Brandt et al. conducted a study in which 12 adults with TS completed a paired associative stimulus (PAS) procedure (64). During this procedure, electrical stimulation is repeatedly administered at the wrist (with a peripheral nerve stimulator) and over primary motor cortex (M1) (with a TMS coil) such that both stimuli simultaneously arrive in the cortex, which, theoretically, should transiently strengthen involved synapses (64). Motor evoked potentials (MEPs) were measured using EMG at the right abductor pollicis brevis muscle. The change in MEP from pre- to post-PAS procedure strongly correlated with PUTS score, suggesting greater sensorimotor cortex plasticity was associated with greater premonitory urge severity. Interestingly, although greater long-term potentiation-like response was associated with greater premonitory urge severity, MEP change was lower in those with TS compared to controls. In a sample of 17 children with TS, Larsh et al. found that higher I-PUTS intensity score (and lower YGTSS-TTS) was associated with lower TMS-evoked cortical excitability and lower long-interval cortical inhibition in the left and right M1 (67). Batschelett et al. used TMS and EMG to assess measures of M1 physiology, including short-interval cortical inhibition and intracortical facilitation, in a sample of 30 children with TS (68). They found no significant associations between M1 physiology measures and I-PUTS scores.

## 4 Discussion

In this scoping review, we identified 155 publications of studies examining the assessment, characteristics, clinical correlates, neural correlates, and treatment of premonitory urges. The extant literature reveals important knowledge and knowledge gaps concerning our current understanding of premonitory urges.

Several measures exist to quantify premonitory urge, but the PUTS is by far the most frequently utilized and has been translated into multiple languages. The PUTS has exhibited acceptable (13) internal consistency in adolescent and adult tic disorder populations (14, 15, 19, 24), with more varied internal consistency in tic disorder populations younger than 10-years-old (12, 18, 20–22). Factor analyses of the PUTS have yielded inconsistent results in terms of items that load onto particular factors, although many of these analyses proposed similar names for factors (“quality,” “intensity”), and across multiple studies items 4 (“not just-right”) and 5 (“something isn’t complete”) loaded onto a factor interpreted as “OCD-related items” or “just right phenomena” (17, 22, 24, 26). The mixed results of PUTS factor analyses may be partially explained by differences in items included in the analyses (i.e., 9-item vs 10-item PUTS), version language (e.g., English, Spanish), and sample population characteristics (e.g., age, prevalence and severity of psychiatric comorbidities, sample size). Further research is needed to clarify the dimensionality, test-retest reliability [as intraclass correlation may be more suitable than Pearson’s correlation in this context (13)], and floor and ceiling effects of the PUTS. The PUTS is a brief, easily administered and scored method for assessing premonitory urge, but the scale has notable limitations: the PUTS produces a single score, reducing premonitory urge to a unidimensional construct, with no clear differentiation between quality, frequency, and intensity of urges; the PUTS score significantly correlates with various internalizing symptoms, including anxiety and obsessive-compulsive symptoms, potentially indicating the scale’s discriminant validity may be suboptimal; and the PUTS provides no explicit timeframe for respondents to consider when answering questions about urges. The clinician-administered I-PUTS, while more time-intensive, addresses many of these limitations, generating details about individual premonitory urges, quantifying premonitory urge in three separate dimensions, and specifying the timeframe in question. While assessed in only two studies thus far, the I-PUTS has demonstrated good internal consistency and inter-rater reliability, as well as potentially superior divergent validity to the PUTS (33, 34). Further research is needed to replicate these findings and to clarify the scale’s floor and ceiling effects. Notably, the I-PUTS, unlike the PUTS, does not assess the quality of premonitory urge. English versions of the PUTS and I-PUTS exhibited small correlations, while Chinese versions of the two scales exhibited moderate correlations. More granular assessment of urge intensity is possible with live urge monitor and urge thermometer approaches, though as typically implemented, these temporally refined approaches do not provide information about different types of urges experienced and their associations with particular tics. Each of the existing methods for assessing premonitory urge possesses advantages and disadvantages, and context will determine which tool is most appropriate. One important direction for future research is to develop methods that capture the valence and quality of premonitory urge. Existing measures do not directly address how uncomfortable or painful urges are; rather, they assess the degree or intensity of urges. Additionally, the aforementioned scales either make no mention of urge quality or assess urge quality by providing a relatively short list of qualities to endorse that may not adequately correspond to an individual’s urge experience. Limited research exists on the subjective qualities of premonitory urges (3, 54). Ultimately, accurate characterization and quantification of premonitory urge dimensions are critical to identifying clinical and neurobiological correlates, developing targeted interventions, and gauging evolution and response of the phenomenon over time.

Premonitory urges are prevalent in tic disorder populations. Though estimates vary widely, many studies observed prevalence rates greater than 60% (17, 50), with some observing rates greater than 90% (48). Variability in prevalence estimates likely arises in part due to differences in study methods for premonitory urge ascertainment [e.g., multi-part questionnaire about urge (17) versus binary question about urge presence/absence (50)] and due to differences in sample population characteristics (e.g., child, adolescent, and/or adult participants). Multiple cross-sectional studies involving large sample populations have observed that prevalence of urges increases from childhood to adolescence (17, 43, 50), with urges typically emerging a few years after tic onset (48). However, evidence for a relationship between severity of urges (based on PUTS scores) and age is mixed. Longitudinal studies, of which there are few to date (21, 69, 88), will be crucial to enhance knowledge of the trajectory of premonitory urges over time.

Most studies exploring the subjective quality of premonitory urges have employed questionnaires, as opposed to qualitative methods (17, 19, 52, 53). From these largely quantitative approaches, overarching characteristics of premonitory urges emerge. Bodily tension and restlessness appear to be the most frequently endorsed quality of urges (17, 19, 52, 53). A “not-just-right” urge quality is more common among individuals with comorbid OCD (19, 47, 53). The majority of adults and children with tics experience more than one distinct premonitory urge (33, 48), though some do not experience urges with every tic and/or every time a given tic occurs (47). Premonitory urges most commonly manifest as localized bodily sensations in the muscles near the site of the associated tic (44, 47, 48, 50, 56), with urge intensity tending to increase immediately prior to the tic (35–37, 63). Most individuals experience relief from their urges following tic execution (19, 44, 47, 48). The distress due to urges has been examined in only two studies, both observing that a subset of individuals experience urges as bothersome in and of themselves (3, 55). More severe premonitory urges are associated with greater disability, poorer global functioning, and poorer health-related quality of life (37, 53, 58, 59, 65, 66, 82, 87, 88), but the degree to which premonitory urges causally contribute to these negative outcomes remains uncertain.

The mechanistic and developmental relationship between urges and tics is not clear, but logically one would expect a significant association between real-time urge intensity and tic occurrence, and this, indeed, is the case (15, 35–37). However, correlations between the PUTS and YGTSS show mixed results, with many studies showing weak or non-significant correlations, possibly in part due to limitations of these scales. Two studies have found a significant relationship between premonitory urges and negative consequences related to tics (e.g., bullying) (28, 72), leading the authors to speculate that negative consequences of tics cause pre-tic sensations to become more aversive. In the only study to assess the longitudinal relationship between premonitory urge and tic severity, baseline PUTS score did not predict tic severity or impairment at an average follow-up time of 11 years; however, participants with more severe premonitory urges at baseline demonstrated less reduction in tic severity at follow-up (69). Importantly, tic and premonitory urge severity are differentially affected by some interventions [e.g., aripiprazole (84), ecopipam (100), MNS (123), CBIT (95), and ERP (97)], possibly indicating that urge and tic severity can be decoupled, though differences in the properties of the scales [e.g., responsiveness (13)] used to quantify these phenomena are also an important consideration. Additionally, some (43, 50), but not all (24, 70, 71), studies suggest that the presence of premonitory urge enhances tic suppression ability. Deeper insights into the relationship between premonitory urges and tics may facilitate development of novel interventions for both symptoms.

Of the comorbid psychiatric features of tic disorders, premonitory urge severity is most commonly associated with OCD symptom severity. Studies have found greater intensity (19) and prevalence (47, 53) of the “not just right” quality of urge in those with comorbid OCD or obsessive-compulsive symptoms. PUTS items 4 (feeling that “something is not ‘just right’”) and 5 (feeling that “something isn’t complete”) often load onto a factor interpreted as “OCD- related items,” and these two items may drive the association between premonitory urge severity and OCD symptom severity. In one study of 656 youth, correlations between PUTS score and OCD measures were no longer significant after removing PUTS items 4 and 5 (17). Given its relatively consistent association with OCD symptoms, investigations of premonitory urge should account for the presence and/or severity of these symptoms in their study design and/or analyses.

Premonitory urges are a sensory phenomenon, and thus one would assume that urges are associated with alterations in interoceptive and/or exteroceptive processing. To date, however, investigations into the relationships between perceptual processes and premonitory urges have generated mixed results. Considering self-reported perceptual experience, PUTS score correlated with interoceptive sensibility [“self-reported sensitivity to bodily sensations” (73)] in two studies of adults (73, 75) but not a third (74) and correlated with sensory hypersensitivity [“heightened awareness of and reactivity to external stimuli” (31)] in one study of adults (31) but not two others involving both children and adults (61, 78). Conflicting results may have arisen due to differences in questionnaires, sample population ages and clinical characteristics, and/or sample sizes. Considering objectively quantified sensory processing, PUTS score correlated with embodiment prediction error (“mismatch of subjective and objective embodiment”) in one study of adults with TS (77) and correlated with interoceptive accuracy, as determined by heartbeat counting tasks, in one study of adults (76) but not in another study of adults (73) nor in two studies of youth (27, 30). PUTS score did not significantly correlate with any parameters in a quantitative sensory testing battery administered to a small sample of adults (52). The lack of identified associations may be due to true absence of a relationship of objective interoception and exteroception measures with premonitory urge, but further investigations are warranted given biological plausibility of such relationships. Anatomical structures involved in interoception [e.g., insular cortex (147)] and exteroception [e.g., somatosensory cortex (148)] are abnormal in individuals with tic disorders, and moreover, several of these same structures are implicated in the pathophysiology of premonitory urge, as will be discussed subsequently.

A number of studies, many with small sample sizes, identified interventions that improved premonitory urge severity, including “urge acceptance” (91), botulinum toxin injections (103–107), topiramate (101), DBS (117–119), and repetitive TMS (124). However, of the botulinum toxin studies, only one was a randomized, double blind, placebo-controlled trial (105), and none of the botulinum toxin studies nor the topiramate study used a validated scale to assess premonitory urges. Of the DBS studies (117–120), the sole randomized, double blind trial comparing thalamic, GPi, and sham stimulation showed no effect of DBS on urges (120). In a sham-controlled, randomized controlled trial of 30 adolescents and adults, repetitive TMS to the bilateral parietal cortices significantly improved premonitory urges and tics, with effects lasting through the end of the trial (one month) (124), but further research is needed to replicate these findings and determine duration of improvement. Unfortunately, common behavioral therapies (CBIT, ERP, HRT) generally did not impact premonitory urge severity [**Supplementary Table S10** (92, 94, 95, 97, 98)]. Interestingly, one session of HRT combined with D-cycloserine improved premonitory urge frequency, but not intensity, more than one session of HRT alone (102). MNS did not significantly improve premonitory urges in a double blind, sham-controlled trial (123). Although several promising interventions were identified in this review, key limitations existed for many studies, including small sample sizes, lack of blinding, lack of a control arm, and/or unvalidated methods for measuring premonitory urges. Furthermore, given that age and psychiatric comorbidity (particularly OCD) are associated with premonitory urge severity, clinical trials should account for these variables in their study design and/or analyses.

Studies that sought to identify anatomical and/or functional correlates of premonitory urge varied in their imaging modalities, experimental tasks, participant characteristics, and statistical approaches, but despite these methodological differences, published findings do implicate specific brain structures, particularly the insula and SMA, in emergence of the premonitory urge. Premonitory urge was associated with differences in insula gray matter volume (57, 139), insula white matter microstructure (143), and functional connectivity of the insula with various other regions in resting state and during a face perception task (125, 126, 133). Two of the three above studies found premonitory urges were associated with functional connectivity between the insula and the SMA. Aspects of the SMA, including its GABA concentration (134) and functional connectivity with basal ganglia structures during voluntary action (132), have been shown to be associated with premonitory urges. The SMA also appears to display heightened activity prior to tics (136, 137), the time period in which urges are present. Physiological differences in other brain regions, including the plasticity of the sensorimotor cortex (64) and the excitably of the motor cortex (67), also seem to be associated with premonitory urges.

The means by which dysfunction in these structures generates the experience of premonitory urge is unclear, but Rae et al. have proposed a theoretical model, based on aberrant predictive processing (149). In this model, GABAergic interneuron dysfunction in the somatosensory region of the putamen leads to transmission of abnormally precise sensory predictions to the primary somatosensory cortex, posterior insula, and anterior insula. The anterior insula must integrate these overly precise sensory predictions with sensory input reflecting current bodily state, resulting in a prediction error that is posited to culminate in a conscious bodily discomfort. Per this same model, the anterior insula also transmits signals to the SMA and other midline motor structures to prompt a corrective movement, i.e., tic, thereby resolving the sensory prediction error. Further research is needed to empirically test this compelling model.

A fully developed mechanistic theory of premonitory urges must also account for several additional facts: premonitory urges become increasingly prevalent with age, not all individuals with tics experience urges, and not all tics within a given individual are associated with urges. Longitudinal studies will be critical to quantify the relationship between premonitory urge and implicated pathophysiologic mechanisms over time. In particular, longitudinal studies following children with tic disorders through early adolescence may yield insights into how urges arise during development and clarify if the alterations in brain structure and function associated with premonitory urges are present and/or change in children who do not endorse that symptom.

Many uncertainties also remain regarding the real-time neural correlates of premonitory urges, as studies to date have either assessed the relationship of a clinical scale (quantifying urge severity over an indefinite period or the past week, i.e., PUTS or I-PUTS, respectively) with a neuroimaging metric or recorded brain activity prior to tics and inferred the presence of urges. No study, to our knowledge, has paired a high temporal resolution neuroimaging technique with a live urge monitor to assess the neural correlates of urge in real time.

## 5 Conclusion

Premonitory urges are prevalent, bothersome features of tic disorders, and, to this point, have received substantially less attention than tics in studies of CTD populations. More research is needed to properly characterize and measure premonitory urge and to clarify its neurobiological basis, which will inform strategies to effectively treat this core feature of tic disorders. Important areas of focus for future research include qualitative and longitudinal studies of premonitory urge, ongoing optimization of premonitory urge measures, further determination of premonitory urge’s responsiveness to established interventions for tics, development of interventions targeting premonitory urge, and elucidation of premonitory urge’s neurobiological underpinnings.

## Data Availability

The original contributions presented in the study are included in the article/**Supplementary Material**. Further inquiries can be directed to the corresponding author.
