# Supplementary material for: Premonitory urge in tic disorders – a scoping review
Source: Front Psychiatry. 2025 Jan 30;16:1504442. doi: 10.3389/fpsyt.2025.1504442 (PMC11821575; doi:10.3389/fpsyt.2025.1504442)
Supplement: Supplementary file 1 [file SupplementaryFile1.docx]

Supplementary Material

# Supplementary Data

## Data Extraction Tool

During the data extraction phase of the review, the tool below was used to extract data from each publication included in the review. The tool was incorporated into Covidence, a systematic review management software (1). For each included publication, two reviewers (J.B.W. and D.A.I.) independently extracted data that fell into one of the categories listed below and recorded this data under the appropriate category in the data extraction tool. After both reviewers completed this process for all included articles, the same two reviewers met to ensure that there was a consensus regarding the data listed under each category for each publication.

1. Study design
2. Cross-sectional versus longitudinal study
3. Observational and/or interventional study
4. Study clinical assessment methods
   1. Method of tic disorder diagnosis (e.g., self-report, clinician diagnosed)
   2. Rating scales used
5. Presence/characteristics of a control group
6. Eligibility criteria and/or participants excluded during the course of the study (e.g. outliers)
7. Clinical characteristics of the target population under study
   1. Number of individuals studied
   2. Age (mean/median, standard deviation / interquartile range, range)
   3. Sex
   4. Race
   5. Ethnicity
   6. Population Setting, i.e., community-based versus clinic-based population
   7. Types of tic disorders included in the target population
   8. Comorbid neuropsychiatric diagnoses
   9. Psychotropic medication use (e.g., percentage taking psychotropic medication)
8. Method(s) of premonitory urge ascertainment
9. Tool used to measure (e.g., self-report scale, urge monitor)
10. Dimensions of urge measured with the tool
11. Quality
12. Location
13. Temporal pattern in relation to tic
14. Intensity
15. Frequency
16. Distress
17. Ability to resist
18. Relief from urge following tic
19. Quantification method
20. Temporal scale for measurement (e.g., momentary assessment of urge, self-report survey inquiring about the past week, etc.)
21. Reported psychometric statistics of rating scales (if relevant)
22. Validation procedure for experimental tools / paradigms (if relevant)
23. Rating of premonitory assessment tool by participants (e.g. Did this questionnaire accurately capture your symptoms?)
24. Associations/statistical relationships between different measures of premonitory urge
25. Characteristics of premonitory urge
26. Method of ascertainment used to identify below characteristics for given study
27. Qualitative characteristics
    - 1. Quality
      2. Location
      3. Temporal pattern in relation to tic
      4. Intensity
      5. Frequency
      6. Distress
      7. Ability to resist
      8. Relief from urge following tic
28. Quantitative characteristics
    - 1. Temporal pattern in relation to tic
      2. Intensity
      3. Frequency
      4. Distress
      5. Abilify to resist
      6. Relief from urge following tic
29. Associations between premonitory urge and clinical characteristics
30. Particular clinical characteristic (e.g., tic intensity, health-related quality of life, age)
31. Method for measuring clinical characteristic (e.g., YGTSS, GTS-QOL)
32. Tool used to measure premonitory urge
33. Statistic used to assess association
34. Finding of association and statistical significance
35. Premonitory urge’s response to treatment and suppression
36. Response to suppression
37. Instructions for suppression
38. Length/frequency/number of suppressions
39. Measurement tool for urge
40. Frequency/number of assessments of urge
41. Length of time between initial assessment of urge and final assessment of urge
42. Presence and characteristics of a control group/condition
43. Changes in urge measurement scores during suppression and following suppression, or differences between urge scores across groups/conditions
44. Relationship between changes in urge measurement scores and changes in clinical characteristics
45. Response to treatment
46. Type of treatment
47. Length/frequency/number of treatment administrations
48. Measurement tool for urge
49. Frequency/number of assessments of urge
50. Length of time between initial assessment of urge and final assessment of urge
51. Presence and characteristics of a control group/condition
52. Changes in urge measurement scores following treatment, or differences in urge scores across group/condition
53. Relationship between changes in urge measurement scores and changes in clinical characteristics
54. Neural correlates of premonitory urge / methods for investigating neural correlates of premonitory urge
55. Tool used to measure premonitory urge
56. Modality for measuring neural structure/function (e.g. fMRI)
57. Instructions for individuals while neural activity is being recorded
58. Temporal precision of urge measurement in relation to neural measurement (e.g. live urge monitoring during fMRI, comparing PUTS to fMRI)
59. Presence/characteristics of different groups/conditions
60. Statistic used to assess association between premonitory urge and neural correlate, or differences between groups/conditions
61. Finding of association and statistical significance

9. Other relevant information

## Search Strategies

**PubMed**

(premonitory[tiab] OR "premonitory urge*"[tiab] OR "premonitory feeling*"[tiab] OR "premonitory sensation*"[tiab] OR "urge-tic network"[tiab] OR "urge to tic"[tiab] OR "tic urge*"[tiab] OR "urge phenomen*"[tiab] OR "associated urge*"[tiab]) AND (Tics[MeSH] OR tic[tiab] OR tics[tiab] OR "habituation spasm*"[tiab] OR "habit chorea*"[tiab] OR "habit spasm*"[tiab] OR Tic Disorders[MeSH] OR "tic disorder*"[tiab] OR Tourette Syndrome[MeSH] OR "Tourette syndrome"[tiab] OR Tourette*[tiab])

**Embase**

(premonitory:ab,ti OR 'premonitory urge*':ab,ti OR 'premonitory feeling*':ab,ti OR 'premonitory sensation*':ab,ti OR 'urge-tic network':ab,ti OR 'urge to tic':ab,ti OR 'tic urge*':ab,ti OR 'urge phenomen*':ab,ti OR 'associated urge*':ab,ti) AND ('tic'/exp OR 'tic*':ab,ti OR 'habituation spasm*':ab,ti OR 'habit chorea*':ab,ti OR 'habit spasm*':ab,ti OR 'tic disorder*':ab,ti OR 'gilles de la tourette syndrome'/exp OR 'tourette syndrome':ab,ti OR 'tourette*':ab,ti)

**PsycINFO**

(tiab(premonitory) OR tiab("premonitory urge*") OR tiab("premonitory feeling*") OR tiab("premonitory sensation*") OR tiab("urge-tic network") OR tiab("urge to tic") OR tiab("tic urge*") OR tiab("urge phenomen*") OR tiab("associated urge*")) AND (MAINSUBJECT.EXACT("Tics") OR MAINSUBJECT.EXACT("Tourette Syndrome") OR tiab(tic*) OR tiab("habituation spasm*") OR tiab("habit chorea*") OR tiab("habit spasm*") OR tiab("Tourette syndrome") OR tiab("Tourette*"))

**Web of Science**

(TI=(premonitory) OR AB=(premonitory) OR TI=("premonitory urge*") OR AB=("premonitory urge*") OR TI=("premonitory feeling*") OR AB=("premonitory feeling*") OR TI=("premonitory sensation*") OR AB=("premonitory sensation*") OR TI=("urge-tic network*") OR AB=("urge-tic network*") OR TI=("urge to tic") OR AB=("urge to tic") OR TI=("tic urge") OR AB=("tic urge") OR TI=("urge phenomen*") OR AB=("urge phenomen*") OR TI=("associated urge*") OR AB=("associated urge*")) AND (TI=("tic*") OR AB=("tic*") OR TI=("habituation spasm*") OR AB=("habituation spasm*") OR TI=("habit chorea*") OR AB=("habit chorea*") OR TI=("habit spasm*") OR AB=("habit spasm*") OR TI=("Tourette syndrome") OR AB=("Tourette syndrome") OR TI=("Tourette*") OR AB=("Tourette*"))

**Scopus**

(TITLE-ABS({premonitory} OR {premonitory urge} OR {premonitory urges} OR {premonitory feeling} OR {premonitory feelings} OR {premonitory sensation} OR {premonitory sensations} OR {urge-tic network} OR {urge to tic} OR {tic urge} OR {tic urges} OR {urge phenomenon} OR {urge phenomena} OR {associated urge} OR {associated urges})) AND (TITLE-ABS({tic} OR {tics} OR {habituation spasm} OR {habituation spasms} OR {habit chorea} OR {habit choreas} OR {habit spasm} OR {habit spasms} OR {tic disorder} OR {tic disorders} OR {Tourette Syndrome} OR {Tourette} OR {Tourettes}))

# Supplementary Tables

Table 1: Psychometric Properties of the Premonitory Urge for Tics Scale (PUTS)

| **Study** | **Psychometric Properties of Interest** | **Sample Size/Demographics** | **Key findings** |
| --- | --- | --- | --- |
| Woods et al (2005) (initial development and validation study) (2) | Internal consistency (Cronbach’s alpha) for 9-item PUTS (English)  Test-retest reliability of 9-item PUTS at one and two weeks | 42 children and adolescents with CTDs | α = 0.81 for entire sample  α = 0.57 for subgroup ≤ 10 years old  α = 0.89 for subgroup > 10 years old  Test-retest reliability (correlation type unclear)  one week: r = 0.79, p < .01  two weeks: r = 0.86, p < .01  one week for subgroup ≤ 10 years old: r = 0.75, p < .01  two week for subgroup > 10 years old: r = 0.81, p < .01 |
| Steinberg et al (2010) (3) | Internal consistency (Cronbach’s alpha) for 9-item PUTS (Hebrew) | 40 children with CTDs | α = 0.79 for entire sample  α = 0.69 for subgroup ≤ 10 years old  α = 0.83 for subgroup > 10 years old |
| Gulisano et al (2015) (4) | Internal consistency (Cronbach’s alpha) for 9-item PUTS (Italian) | 95 children with TS assessed at baseline and long-term follow up | α = 0.70 for subgroup ≤ 10 years old  α = 0.85 for subgroup > 10 years old |
| Raines et al (2018) (5) | Internal consistency (Cronbach’s alpha) for 9-item PUTS (English)  EFA of items 1-8 of PUTS | 84 individuals with CTDs, aged 5-21 | α = 0.82 for entire sample  α = 0.66 for subgroup ≤ 10 years old  α = 0.87 for subgroup > 10 years old  two factors:   1. “specific urge qualities” (items 1, 2, 4, and 5) 2. “a more general urge experience” (items 3, 6, 7, and 8) |
| Forcadell et al (2022) (6) | Internal consistency (Cronbach’s alpha) for 9-item PUTS (Spanish)  Test-retest reliability of 9-item PUTS at four months  EFA of items 2-9 of PUTS | 72 children and adolescents with CTDs (n = 55 for test-retest reliability) | α = 0.88 for subgroup ≤ 10 years old  α = 0.77 for subgroup > 10 years old  r = 0.49, p < .01 entire sample  r = 0.61, p < .01 for subgroup > 10 years old  Correlation not significant for subgroup ≤ 10 years old  two factors:   1. “qualities and frequency of premonitory urges” (items 2, 3, 6, 7, 8, and 9) 2. “mental phenomena related to OCD” (items 4 and 5) |
| Openneer et al (2020) (7) | Internal consistency (Cronbach’s alpha) for 9-item PUTS (Languages unclear)  EFA of items 2-9 of PUTS  EFA of 9-item PUTS with item 2 and item 9 removed in subgroup ≥ 11 years old | 656 children ages 3-16 years old with CTDs | α = 0.84 for subgroup ≤ 7 years old  α = 0.83 for subgroup 8-10 years old  α = 0.76 for subgroup > 10 years old  one factor  two factors:   1. “OCD-related items” (items 4 and 5) 2. “frequency of urges” (items 1, 3, 6, 7, and 8) |
| Li et al (2021) (8) | Internal consistency (Cronbach’s alpha) for 9-item PUTS (Mandarin/Cantonese)  Test-retest reliability of 9-item PUTS at one month  EFA of 9-item PUTS | 367 children with any tic disorder (n = 147 for test-retest reliability) | α = 0.75 for entire sample  α = 0.76 for subgroup < 10 years old  α = 0.73 for subgroup ≥ 10 years old  r = 0.89 (correlation type unclear)  four factors:   1. “intensity” (items 4, 7, 8, and 9) 2. “frequency” (items 2 and 3) 3. “sensory aspect of premonitory urges” (items 5 and 6) 4. “mental aspect of premonitory urges” (item 1) |
| Baumung et al (2021) (9) | Internal consistency (Cronbach’s alpha) for 10-item PUTS (English/German)  EFA of items 1-8 of PUTS | 93 adults with TS and 148 children with TS | α = 0.82 for minors  α = 0.78 for adults  two factors:   1. “intensity or frequency” (items 1, 6, 7, and 8 in adult sample, and items 1, 2, 6, 7, and 8 in minor sample) 2. “quality” (items 2, 3, 4, and 5 in adult sample, and items 3, 4, and 5 in minor sample) |
| Matsuda et al (2020) (10) | Internal consistency (Cronbach’s alpha) for 9-item PUTS (Japanese)  EFA of 9-item PUTS | 62 children and adults with TS | α = 0.85 for entire sample  α = 0.85 for minors  α = 0.87 for adults  three factors:   1. “quality” (items 1, 2, and 3) 2. “intensity” (items 1, 7, and 8) 3. “just right phenomena” (items 4, 5, and 9) |
| Brandt et al (2016) (11) | Internal consistency (Cronbach’s alpha) for 10-item PUTS (German)  EFA of 10-item PUTS | 22 adults with TS, 40 children/adolescents with TS | α = 0.84 for minors  α = 0.79 for adults  three factors:   1. “quality” (items 2, 3, 4, and 5 in adult sample, and 3, 4, and 5 in child sample) 2. “intensity” (items 7 and 8 in adult sample, items 2, 7, and 8 in child sample) 3. “control over tics and urges.” (items 6, 9, and 10 in child and adult samples) |
| Edwards et al (2020) (12) | Internal consistency (Cronbach’s alpha) for 9-item PUTS (English) | 74 individuals aged 5-21 years with CTDs | α = 0.83 for participants with comorbid OCD and/or ADHD  α = 0.69 for adults participants without comorbid OCD or ADHD |
| Zinner et al (2012), Rozenman et al (2015), Reese et al (2014), Pile et al (2018), Kyriazi et al (2019), Kim et al (2020), Crossley et al (2014), Isaacs et al (2020), Schütteler et al (2023) (13–21) | Internal consistency (Cronbach’s alpha) for 9-item PUTS or 10-item PUTS – these studies did not provide a breakdown of internal consistency by age or comorbidity status, and thus are grouped in a single row | Children, adults or both children and adults with TS or CTDs (sample size ranged from 28 to 206) | α ranged from 0.71 (for the 10-item PUTS, 0.75 for the 9-item PUTS in this study (21)) to 0.85 (13,19)) |
| Kim et al (2020) (18) | Test-retest reliability for 9-item PUTS (Korean): interval ranged from two weeks to two months  EFA of 9-item PUTS | 38 children and adults with CTD (n = 20 for test-retest reliability) | Pearson’s r = 0.60  three factors:   1. “presumed functional relationship between the tic and the urge to tic” (items 1, 7, 8, and 9) 2. “the quality of the premonitory urge” (items 2, 3, and 6) 3. “just right phenomena” (items 4 and 5) |
| Reese et al (2014) (15) | Test-retest reliability for 9-item PUTS (English): interval was within 2 weeks | 122 adolescents and adults with CTDs | r = 0.79, p < .001 (correlation type unclear) |

PUTS = Premonitory Urge for Tics Scale, TS = Tourette Syndrome, CTDs = chronic tic disorders, OCD = obsessive-compulsive disorder, ADHD = attention-deficit/hyperactivity disorder, EFA = exploratory factor analysis

Table 2: Associations Between PUTS and Measures of Tics/Tic Disorder Severity

| **Study** | **Associations Investigated** | **Sample Size/Demographics** | **Findings** |
| --- | --- | --- | --- |
| Sutherland-Owens et al (2011) (22) | PUTS scores and YGTSS “global” scores | 18 children and adults with TS, sample age range 10-41 | No significant association found between PUTS scores and YGTSS scores (Simple Regression: r = 0.21, p > 0.05) |
| Gerasch et al (2016) (23) | PUTS scores and YGTSS-TTS / MRVS total scores | 44 adults with TS | No significant association found between PUTS scores and YGTSS-TTS or MRVS total scores (Spearman's r = 0.281, p = 0.065 for YGTSS-TTS, r = 0.042, p = 0.79 for MRVS total scores) |
| Rajagopal and Cavanna (2014) (24) | PUTS item scores and YGTSS scores | 108 adults with TS | No significant Spearman’s correlation between any PUTS item score and YGTSS (based on reported values, authors used global YGTSS score (i.e., sum of total tic score and impairment), though this is not explicitly stated in the paper) scores after correction for multiple comparisons |
| Steinberg et al (2013) (25) | PUTS scores and YGTSS-TTS / YGTSS impairment scores | 56 children and adolescents with CTDs | No significant correlation found between PUTS scores and YGTSS-TTS (Pearson’s r = 0.21) or YGTSS impairment scores (Pearson’s r = 0.27)  In a multiple regression predicting YGTSS impairment from PUTS scores and BATS scores, PUTS was not a significant predictor (β = -0.02) |
| Eddy and Cavanna (2014) (26) | PUTS scores and YGTSS-TTS | 100 adolescents and adults aged 17-67 with TS | Significant correlation found between PUTS scores and YGTSS-TTS, but did not survive correction for multiple comparisons (Pearson's r = 0.273, p = 0.042) |
| Ramsey et al (2021) (27) | PUTS scores and YGTSS-TTS / YGTSS impairment scores | 75 children and adolescents with any tic disorder | No significant correlation found between PUTS scores and YGTSS-TTS or YGTSS impairment scores (Pearson's r = 0.10 for YGTSS-TTS, r = 0.02 for YGTSS impairment scores)  Based on structural equation modeling, urge intolerance (a theoretical construct combing items from the PUTS and DTS) did not predict YGTSS-TTS scores (β = −0.34, p = 0.729) |
| Barnea et al (2016) (28) | PUTS scores and number of tics in five different environmental situations | 41 children and adolescents with TS | PUTS scores not a significant predictor of number of tics in any environmental situation in multiple regressions that included FAI as a covariate |
| Jackson et al (2020) (29) | PUTS scores and YGTSS-TTS / YGTSS impairment scores | 28 participants aged 9-22 with TS | No significant Pearson’s correlations or simple linear regression associations found between PUTS scores and YGTSS-TTS (simple linear regression R^2^ = 0.035, p = 0.34) or YGTSS impairment scores (R^2^ = 0.09, p = 0.13) |
| Müller-Vahl et al (2014) (30) | PUTS scores and MRVS Total Tic Score (TTS) / YGTSS scores | 22 adults with TS | No significant correlations found between PUTS scores and MRVS Total Tic Score or any YGTSS score (Pearson's r value not reported, p > 0.05) |
| Isaacs et al (2021) (31) | PUTS scores and YGTSS-TTS | 52 adults with CTDs | No significant correlation between PUTS scores and YGTSS-TTS after correcting for multiple comparisons (Spearman’s r = 0.34) |
| Narapareddy et al (2022) (32) | PUTS scores and MAIA-2 Not-Worrying scores, MAIA-2 Not-Distracting scores, MAIA-2 composite variable scores, DOCS scores, YGTSS-TTS | 48 adults with CTDs | No significant correlation between PUTS scores and YGTSS-TTS after correcting for multiple comparisons (Spearman’s r = 0.28)  In a multivariable linear regression predicting PUTS scores from MAIA-2 Not-Worrying scores, MAIA-2 Not-Distracting scores, MAIA-2 composite variable scores (i.e., mean score from the six MAIA-2 subscales besides Not-Worrying and Not-Distracting), DOCS scores, and YGTSS-TTS, YGTSS-TTS was a significant predictor of PUTS scores (β = 0.08, p < 0.05). |
| Ganos et al (2012) (33) | PUTS and YGTSS scores | 15 adults with TS | No significant correlation between PUTS scores and YGTSS global score (sum of total tic score and impairment) (Pearson’s r = 0.386, p = 0.155), or any of the YGTSS subscale scores except for the interference subscale (Pearson’s r = 0.546, p = 0.035) |
| Rozenman et al (2015) (14) | PUTS scores and YGTSS-TTS / YGTSS impairment scores | 124 children and adolescents with CTDs | Significant correlation found between PUTS scores and YGTSS-TTS (r = 0.27, p = 0.003), and between PUTS scores and YGTSS impairment scores (r = 0.27, p = 0.002)  YGTSS impairment scores were a significant predictor of PUTS scores in a multiple regression that included SCARED-C Panic/Somatic subscale scores and age as independent variables (β = 0.21, p < 0.01) |
| Kano et al (2015) (34) | PUTS scores and YGTSS-TTS / YGTSS impairment scores | 41 children and adults with TS aged 12-50 | Significant correlations found between PUTS scores and YGTSS-TTS score (Pearson’s r = 0.51, p < 0.001) and YGTSS impairment scores (Pearson’s r = 0.60, p < 0.001) |
| Li et al (2021) (8) | PUTS and YGTSS | 367 children with any tic disorder | Significant correlation found between PUTS scores and YGTSS scores (Pearson’s r = 0.180, p < 0.05) (The specific YGTSS score used is unclear based on the publication)  In the subgroup with TS (n = 252), this correlation became slightly stronger (Pearson’s r = 0.269, p < 0.05) |
| Matsuda et al (2020) (10) | PUTS and YGTSS-TTS | 62 children and adults with TS aged 10-53 | Significant correlation between PUTS scores and YGTSS-TTS scores (Spearman’s r = 0.32, p = 0.04) |
| Ganos et al (2015) (35) | PUTS and YGTSS | 19 adults with TS | In a multiple regression to predict PUTS scores from YGTSS (based on reported values, authors used global YGTSS score (i.e., sum of total tic score and impairment), though this is not explicitly stated in the paper) scores, Y-BOCS scores and interoceptive accuracy performance on a behavioral task, YGTSS scores were a significant predictor (β = 0.6519, p = 0.049) |
| Draper et al (2016) (36) | PUTS and YGTSS motor/vocal tic severity | 29 participants aged 8-21 with TS | Significant correlation between PUTS scores and both YGTSS motor tic severity (Pearson’s r = 0.53, p < 0.002) and YGTSS vocal tic severity scores (Pearson’s r = 0.32, p < 0.05) |
| Gu et al (2020) (37) | PUTS and YGTSS-TTS / YGTSS impairment / YGTSS motor/vocal tic severity | 252 children and adolescents with CTDs | PUTS scores correlated with YGTSS-TTS (Spearman’s r = 0.128, p = 0.043), YGTSS impairment (Spearman’s r = 0.163, p = 0.009), YGTSS motor tic score (Spearman’s r = 0.144, p = 0.022) but not YGTSS vocal tic score (Spearman’s r = 0.081, p = 0.202)  PUTS scores were significantly associated with YGTSS-TTS scores in a simple linear regression (β = 0.174, p = 0.006) |
| Kim et al (2020) (18) | PUTS and YGTSS-TTS / YGTSS impairment | 38 children and adults aged 10-44 with CTDs | PUTS scores significantly correlated with YGTSS-TTS scores (Pearson’s r = 0.55, p < 0.001) and YGTSS impairment scores (Pearson’s r = 0.43, p < 0.01) |
| Reese et al (2014) (15) | PUTS and YGTSS-TTS / YGTSS motor/vocal tic severity | 122 adolescents and adults with CTDs | PUTS scores significantly correlated with YGTSS-TTS scores (r = 0.32, p < 0.001) (correlation type unclear from publication)  PUTS scores significantly correlated with YGTSS vocal tic severity (r = 0.30, p = 0.001) but not motor tic severity (p > 0.05) (correlation type unclear from publication) |
| Ramsey et al (2022) (38) | PUTS/urge intolerance and YGTSS-TTS / YGTSS impairment | 80 adults with TS or chronic motor tic disorder | Significant correlations between PUTS scores and both YGTSS severity (r = 0.37, p = 0.002) and YGTSS impairment (r = 0.43, p < 0.001) (correlation type unclear from publication)  Created a bifactor structural model of urge intolerance (a theoretical construct composed of items within the PUTS and DTS, higher values indicate greater urge intolerance) with premonitory urge and distress tolerance as subfactors to predict YGTSS-TTS and YGTSS impairment - Urge intolerance and both subfactors were significant predictors of both YGTSS severity (Urge intolerance: β = 0.35, p = 0.001, Premonitory urges: β = 0.23, p < 0.05) and impairment (Urge intolerance: β = 0.32, p = 0.005, Premonitory urges: β = 0.34, p < 0.05) |
| Capriotti et al (2013) (39) | PUTS and PTQ | 118 children and adolescents with CTDs | Significant correlation between PUTS and PTQ (Pearson’s r = 0.21, p = 0.03) |
| Brandt et al (2023) (40) | PUTS and YGTSS-TTS / ATQ scores | 111 adults with TS | Significant correlations found between PUTS scores and both YGTSS-TTS (Spearman’s r = 0.20, p < 0.001) and ATQ scores (r = 0.34, p < 0.001) |
| Woods et al (2005) (2) | PUTS scores vs. YGTSS-TTS scores | Children older than 10 years old (n = 23), and ≤10 years (n = 19) with CTDs | Significant correlation between PUTS and YGTSS-TTS scores in participants older than 10 (Pearson’s r = 0.52, p < 0.05), but not significant in participants 10 years and younger (Pearson’s r = -0.25, p > 0.05). |
| Raines et al (2018) (5) | PUTS scores vs. PTQ | Children/young adults older than 10 years old (n = 50), and children ≤10 years (n = 34) with CTDs | PUTS scores correlated with PTQ number of tics subscale in both age groups (≤10 years: Pearson’s r = 0.38, p < 0.05; >10 years: Pearson’s r = 0.31, p < 0.05). No other PTQ subscale significantly correlated with PUTS scores in either subgroup. |
| Steinberg et al (2010) (3) | PUTS scores vs. YGTSS-TTS scores | Children older than 10 years old (n = 22), and  ≤10 years (n = 18) with CTDs | PUTS scores did not correlate with YGTSS-TTS scores in participants ≤10 years (r = -0.09) or >10 years (r = 0.04) (correlation type unclear from publication) |
| Kyriazi et al (2019) (17) | PUTS scores vs. YGTSS-TTS scores | Children aged 6-11 (n = 39), and 12 years and older (n = 13) with any tic disorder | Multiple regression showed PUTS scores predicted YGTSS-TTS scores in the entire sample (β = 0.466, p < 0.001). Significant correlation between YGTSS-TTS and PUTS scores in children aged 6-11-years-old (r = 0.37, p = 0.022) and 12 years and older (r = 0.65, p = 0.016) (correlation type unclear from publication) |
| Openneer et al (2020) (7) | PUTS scores vs. YGTSS-TTS scores | Children ≤7 years (n = 103), 8-10 years (n = 253), >10 years (n = 300) with CTDs | Significant correlation between PUTS and YGTSS-TTS scores in children aged 8-10 years (Pearson’s r = 0.260, p < 0.001), but not in the youngest (Pearson’s r = 0.027) or oldest groups (Pearson’s r = 0.086). |
| Gulisano et al (2015) (4) | PUTS scores vs. YGTSS scores | 95 children with TS at baseline (aged 4-10) and again follow-up (aged 10-16) | No significant correlation between YGTSS scores (unclear which YGTSS score based on publication) and PUTS scores at baseline (Spearman’s r = -0.156, p = 0.131) or follow-up (Spearman’s r = 0.158, p = 0.127). |
| Kano et al (2020) (41) | PUTS scores vs. YGTSS scores | 20 individuals aged 17-53 with TS (participants had been assessed 4.0 years prior on average) | Significant correlations between PUTS and YGTSS scores at baseline (p < 0.001) and follow-up (p = 0.024). (correlation magnitudes not reported in the publication) |
| Brandt et al (2016) (11) | PUTS 9 and PUTS 10 scores vs. YGTSS motor tic scores | 22 individuals aged 17-55 with TS | Significant Pearson’s correlations between YGTSS motor tic severity and both PUTS 10 (r = 0.43, p = 0.048) and PUTS 9 (r = 0.48, p = 0.025) |
| Brandt et al (2014) (42) | PUTS and YGTSS-TTS | 12 adults with TS | Significant correlation between PUTS and YGTSS-TTS (r   = 0.63, p = 0.038) (correlation type unclear from publication) |

TS = Tourette Syndrome, CTDs = chronic tic disorders, YGTSS = Yale Global Tic Severity Scale, TTS = Total Tic Score, MRVS = Modified Rush Video-Based Tic Rating Scale, BATS = Beliefs About Tics Scale, DTS = Distress Tolerance Scale, FAI = Functional Assessment Interview, DOCS = Dimensional Obsessive-Compulsive scale, MAIA-2 = Multidimensional Assessment of Interoceptive Awareness, Version 2, SCARED-C = Screen for Child Anxiety Related Emotional Disorders - Child Version, PTQ = Parent Tic Questionnaire, PUTS = Premonitory Urge for Tics Scale, ATQ = Adult Tics Questionnaire, Y-BOCS = Yale Brown Obsessive Compulsive Scale

Table 3: Associations Between I-PUTS and Measures of Tics/Tic Disorder Severity

| **Study** | **Associations Investigated** | **Sample Size/Demographics** | **Statistical Methods and Findings** |
| --- | --- | --- | --- |
| McGuire et al (2016) (43) | I-PUTS (intensity, number, frequency) and YGTSS-TTS / YGTSS Impairment | Data from 73 children with any tic disorder | YGTSS-TTS significantly correlated with I-PUTS intensity (Spearman’s r = 0.23, p < 0.05) but not with number (Spearman’s r = 0.18) or frequency (Spearman’s r = 0.21). No correlation found between any I-PUTS subscale and YGTSS impairment (all absolute values for r < 0.05).  *In this study, PUTS scores did not significantly correlate with YGTSS-TTS or YGTSS impairment |
| Larsh et al (2022) (44) | I-PUTS intensity and YGTSS-TTS | 17 children with TS | Partial Spearman correlation adjusting for age: Strong correlation found between I-PUTS intensity and YGTSS-TTS scores (r = 0.74, p < 0.01). |
| Batschelett et al (2023) (45) | I-PUTS urge frequency / intensity and YGTSS-TTS | 30 children with TS | YGTSS-TTS significantly correlated with I-PUTS urge frequency (Spearman’s r = 0.721, p = 0.001) and intensity (Spearman’s r = 0.593, p = 0.009). |
| Che et al (2023) (46) | I-PUTS / PUTS and YGTSS-TTS | 123 children with tic disorders | Significant correlations between YGTSS-TSS and all 3 I-PUTS dimensions (Spearman’s r = 0.301, 0.370, 0.339 for number, frequency and intensity respectively, all p < 0.01) as well as between the PUTS and YGTSS-TSS (Spearman’s r = 0.427, p < 0.01). |

I-PUTS = Individualized Premonitory Urge for Tics Scale, PUTS = Premonitory Urge for Tics Scale, TS = Tourette Syndrome, YGTSS = Yale Global Tic Severity Scale, TTS = Total Tic Score

Table 4: Tic Disorder Severity/Characteristics and Premonitory Urges

| **Study** | **Associations Investigated** | **Sample Size/Demographics** | **Statistical Methods and Findings** |
| --- | --- | --- | --- |
| Li et al (2022) (47) | PUTS scores and treatment refractory status | 126 children with TS | Independent samples t-test: Treatment refractory group (n = 64) had significantly higher PUTS scores than non-treatment refractory group (n = 62) (t = 14.61, p < 0.001). |
| Sambrani et al (2016) (48) | Premonitory urge presence and tic severity | 1032 participants with any tic disorder | Independent samples t-test: Participants experiencing premonitory urges had more severe tics (mean STSS-GSR = 2.87) compared to those who did not experience premonitory urges (mean STSS-GSR = 2.62) (t = -3.164, p < 0.005). |
| Eapen and Robertson (2015) (49) | Presence of premonitory urges and presence of complex tics | 400 children and adults with TS – further analyses were conducted on the subset of 222 individuals with TS who had full data on comorbidities and associated psychopathologies | Fisher’s exact test: Participants experiencing complex tics were more likely to report premonitory urges than those without complex tics (φ = 0.15, p = 0.006). |
| Kaczyńska and Janik (2021) (50) | Presence of premonitory urges and presence of tonic tics | Data from 232 children and adults with TS | Chi-square test: Patients with tonic tics were more likely to experience premonitory urges compared to those without tonic tics (p = 0.013). Multivariate logistic regression showed that premonitory urges were a significant predictor of tonic tics (OR = 4.69, p = 0.018), controlling for sex and age. |

STSS-GSR = Shapiro Tourette-Syndrome Severity Scale Global Severity Rating, OR = odds ratio, TS = Tourette syndrome, PUTS = Premonitory Urge for Tics Scale

Table 5: Associations with Obsessive-Compulsive Symptoms/Disorders

| **Study** | **Sample Size/Demographics** | **Findings** |
| --- | --- | --- |
| Crossley and Cavanna (2013) (51) | 72 adolescents and adults with TS | Significant positive correlation between OCI-R scores and PUTS scores (Spearman’s r = 0.38, p = 0.001) |
| Eddy and Cavanna (2014) (26) | 100 adults and adolescents with TS | Significant positive correlation between PUTS scores and OCI-R scores (Pearson’s r = 0.321, p = 0.002), OCI-R scores were significant predictor of PUTS scores in a stepwise linear regression (R^2^ = 0.129, p = 0.012) |
| Matsuda et al (2020) (10) | 62 children and adults with TS | Significant positive correlation between PUTS scores LOI-CV scores (Spearman’s r = 0.52, p < 0.001) |
| Narapareddy et al (2022) (32) | 48 adults with CTDs | Significant positive correlation between DOCS scores and PUTS scores (Spearman’s r = 0.53, p < 0.001), DOCS scores significantly predicted PUTS scores in a multivariable linear regression (β = 0.14, p < 0.05) |
| Gerasch (2016) (23) | 44 adults with TS | Significant positive correlation between PUTS scores and Y-BOCS scores (Spearman’s r = 0.34, p = 0.024) |
| Gulisano et al (2015) (4) | 95 children with TS | Significant positive correlation between CY-BOCS scores and PUTS scores at baseline and follow-up, per authors, though correlation and p-values in Table 2 do not clearly reflect this |
| Kano et al (2020) (41) | 20 individuals with TS aged 17-53 (participants had been assessed 4.0 years prior on average) | Significant Pearson correlation between 1) PUTS scores at baseline and Y-BOCS at baseline (p = 0.002), 2) PUTS scores at follow-up and Y-BOCS at follow-up (p = 0.002), 3) PUTS scores at baseline and Y-BOCS at follow-up (p = 0.019) (correlation magnitudes not reported in the publication) |
| Kano et al (2015) (34) | 41 children and adults with TS | Significant positive correlation between PUTS scores DY-BOCS total OCS scores (Spearman’s r = 0.55, p < 0.001) and DY-BOCS impairment scores (Spearman’s r = 0.59, p < 0.001) |
| Li et al (2021) (8) | 367 children with tic disorders | CY-BOCS scores did not correlate with PUTS scores in the entire sample (Pearson’s r = 0.108, p > 0.05), but in the subgroup with Tourette syndrome this correlation was significant (n = 252, r = 0.203, p < 0.05) |
| Rajagopal and Cavanna (2014) (24) | 108 adults with TS | Items 1, 3, 4 and 6 of the PUTS correlated with the compulsions subscale of the MOVES (Spearman’s r = 0.305, r = 0.306, r = 0.297, r = 0.341 respectively), and items 2 and 5 correlated with the obsessions and compulsions subscales of the MOVES (PUTS item 2 correlations: r = 0.371 and r = 0.312, respectively; PUTS item 5 correlations: r = 0.287 and r = 0.448, respectively). Items 5 and 6 of the PUTS also showed significant correlations with Y-BOCS scores (both p = 0.001, these correlation magnitudes were not reported in the publication) |
| Brandt et al (2023) (52) | 291 adults with CTDs | Greater OCI-R scores associated with greater urge intensity as assessed on a 1–11-point scale (β = 0.22, p = 0.017) in a linear regression with age, gender, and other scales assessing symptoms for comorbid disorders as covariates |
| Brandt et al (2023) (40) | 111 adults with TS | Significant correlations between PUTS scores and Y-BOCS scores (Spearman’s r = 0.34, p < 0.001), and OC-TCDQ scores (Spearman’s r = 0.35, p < 0.001) |
| Isaacs et al (2021) (31) | 52 adults with CTDs | Non-significant correlation between PUTS scores and DOCS scores after correcting for multiple comparisons (Spearman’s r = 0.36) |
| Ganos et al (2015) (35) | 19 adults with TS | Y-BOCS scores were not a significant predictor of PUTS scores in a multiple regression (β = -0.22, p = 0.50) |
| Rozenman et al (2015) (14) | 124 children and adolescents with CTDs | CYBOCS scores were not a significant predictor of PUTS scores in a multiple linear regression analysis (p > 0.22) |
| Sambrani et al (2016) (48) | 1032 children and adults with any tic disorder | Significant association between presence of premonitory urges and OCB (χ^2^ = 15.38, p < 0.001), obsessions (χ^2^ = 11.22, p < 0.01), and compulsions (χ^2^ = 26.77, p < 0.01), but not OCD (χ^2^ = 3.085, p > 0.05) |
| Sutherland-Owens et al (2011) (22) | 18 children and adults with TS | Non-significant correlations between PUTS scores and the obsessions and compulsions subscales of the CY-BOCS/Y-BOCS (r = -0.28, r = -0.20 respectively, p > 0.05, correlation type unclear from publication) |
| Brandt et al (2016) (11) | 22 adults with TS | Significant correlation between Y-BOCS scores and PUTS-10 scores (Pearson’s r = 0.43, p < 0.05), but not average real-time urge monitor scores (Pearson’s r = 0.11, p > 0.05) or PUTS-9 scores (Pearson’s r = 0.41, p > 0.05) |
| Kim et al (2020) (18) | Data from 20 children and adults with CTDs | Non-significant Spearman’s correlations between PUTS scores and CY-BOCS/Y-BOCS scores (p > 0.05, correlation magnitudes not reported in publication) |
| Reese et al (2014) (15) | 122 adolescents and adults with TS | Non-significant correlation between Y-BOCS total scores and PUTS scores (r = 0.11, p = 0.25) (correlation type unclear from publication) |
| Woods et al (2005) (2) | 42 children and adolescents with CTDs | Positive correlation between PUTS scores and CYBOCS scores (Pearson’s r = 0.31, p < 0.05)  Sample was divided into subgroups aged 8-10 (n = 19) and aged 11-16 (n = 23) - this correlation only remained significant in the older group (older group: r = 0.55, p < 0.01, younger group: r = 0.24, p = 0.34) |
| Steinberg et al (2010) (3) | 40 children with CTDs | CY-BOCS scores correlated with PUTS scores (r = 0.50, p < 0.01) (correlation type unclear from publication). The obsessions subscale of the CY-BOCS was a significant predictor of PUTS scores in a multiple regression (β = 0.40, p < 0.01)  After dividing the sample into subgroups of 10 years and younger and older than 10 years, the correlation between CY-BOCS scores and PUTS scores was significant for the older group (r = 0.61, p < 0.01) but not the younger group (r = 0.17, p > 0.05). |
| Openneer et al (2020) (7) | 656 children with CTDs | Obsessions and compulsions subscale scores of the CY-BOCS each correlated with PUTS scores (Pearson’s r = 0.106, p < 0.001 and Pearson’s r = 0.126, p < 0.001 respectively), despite CY-BOCS total scores not correlating with PUTS scores (r = 0.083, p > 0.05)  After separating the sample into children aged 7 years and under, aged 8-10 years and older than 10 years the correlations of the PUTS with these CY-BOCS subscale scores only remained significant in the group aged 8-10 (r = 0.184, p < 0.001 and r = 0.221, p < 0.001 for obsession and compulsions respectively) |
| Edwards et al (2020) (12) | 74 individuals aged 5-21 with CTDs | PUTS scores correlated with number of tics subscale of the PTQ in those with comorbid OCD and/or ADHD (n = 35, Pearson’s r = 0.47, p < 0.01), but not in those without comorbid OCD and/or ADHD |

YBOCS = Yale-Brown Obsessive-Compulsive Scale, OCI-R = Obsessive-Compulsive Inventory - Revised, LOI-CV = Leyton Obsessional Inventory-Child Version, DOCS = Dimensional Obsessive-Compulsive scale, DY-BOCS = Dimensional Yale-Brown Obsessive-Compulsive Scale, CY-BOCS = Children’s Yale-Brown Obsessive-Compulsive Scale, MOVES = The Motor tic, Obsessions and compulsions, Vocal tic Evaluation Survey, OC-TCDQ = Obsessive–Compulsive Trait Core Dimensions Questionnaire, OCB= Obsessive-compulsive behavior, ADHD = = Attention-Deficit Hyperactivity Disorder, OCD = Obsessive Compulsive Disorder, CTD = Chronic Tic Disorder, PUTS = Premonitory Urge for Tics Scale, TS = Tourette Syndrome, OCS = obsessive-compulsive symptoms

Table 6: Associations with Attention Deficit Hyperactivity Symptoms/Disorders

| **Study** | **Sample Size/Demographics** | **Findings** |
| --- | --- | --- |
| Brandt et al (2016) (11) | 22 adults with TS | No significant correlations between current ADHD symptoms (ADHD-SB) or childhood ADHD symptoms (WURS-K) and premonitory urges (PUTS-9, PUTS-10 scores, or live urge monitor values).  Significant correlations found between childhood ADHD (WURS-K) symptoms and specific items on the PUTS (item 3: Pearson's r = 0.56, p = 0.006; item 4: Pearson's r = 0.46, p = 0.032) as well as between PUTS item 3 and ADHD-SB total scores and ADHD-SB hyperactivity subscale scores (r = 0.44, p = 0.047, r = 0.64, p = 0.002 respectively). |
| Kim et al (2020) (18) | Data from 35 children and adults with CTDs | Significant negative correlation between PUTS and ARS scores in subgroup 15 years and younger (n = 21, Spearman’s r = -0.47, p <0.05) but no significant correlation between PUTS and ASRS scores in adults (p > 0.05) |
| Gerasch et al (2016) (23) | 44 adults with TS | PUTS scores significantly correlated with the attention deficit domain of the DSM-IV symptom list for ADHD (Spearman’s r = 0.313, p < 0.05) and CAARS total scores (r = 0.411, p < 0.05) |
| Langelage et al (2022) (53) | 25 children and adolescents with TS | Significant correlations between intensity of premonitory urges (average intensity of live urge monitor ratings over five minutes) and ADHD symptoms measured by Conners ADHD rating scale (IA subscale: Pearson's r = 0.57, p = 0.01; HI subscale: Pearson's r = 0.50, p = 0.035). |
| Crossley and Cavanna (2013) (51) | 72 adolescents and adults with TS | Significant correlation between ASRS and PUTS scores (Spearman's r = 0.37, p = 0.001). |
| Eddy and Cavanna (2014) (26) | 100 adults and adolescents with TS | Significant correlation between ASRS and PUTS scores (Pearson’s r = 0.283, p = 0.004) |
| Isaacs et al (2021) (31) | 52 adults with CTDs | No significant correlation between ASRS and PUTS scores (Spearman's r = 0.19, p > 0.001). |
| Narapareddy et al (2022) (32) | 48 adults with CTDs | No significant correlation between PUTS and ASRS scores (Spearman's r = 0.28, p-value not reported but above significance threshold established by Benjamini et al’s false discovery rate-controlling procedure.) |
| Steinberg et al (2010) (3) | 40 children with CTDs | PUTS scores did not significantly correlate with ADHD Rating Scale IV (Conners) scores (r = 0.22, p = 0.16) (correlation type unclear from publication)  After dividing the sample into younger (less than 10-years-old) and older (older than 10 years) subgroups, this correlation remained non-significant in both groups |
| Reese et al (2014) (15) | 122 adolescents and adults with CTDs | PUTS scores did not significantly correlate with ADHD-RS scores (r = 0.08, p = 0.39, correlation type unclear from publication) |
| Gulisano et al (2015) (4) | 95 children with TS | No significant correlation between premonitory urges and ADHD symptoms measured by the CADS at baseline (mean age = 7.3 years, SD = 1.5 years) (Spearman's r = -0.156, p = 0.12) or follow-up (mean age = 13.1 years, SD = 3.7 years) (Spearman's r = 0.162, p = 0.13). |
| Openneer et al (2020) (7) | 656 children with CTDs | Weak but significant correlation between ADHD symptom severity (measured by SNAP-IV) and premonitory urges (Pearson's r = 0.080, p < 0.05).  After separating the sample into children aged 7 years and under, aged 8-10 years and older than 10 years the correlation only remained significant for the group aged 8-10 years (Pearson's r = 0.140, p < 0.05). |

ADHD = Attention-Deficit Hyperactivity Disorder, ADHD-SB = German ADHD self-rating scale, WURS-K = Wender Utah Rating Scale, DSM-IV = Diagnostic and Statistical Manual of Mental Disorders, fourth edition, CAARS = Conners Adult ADHD Rating Scale, IA = Inattention subscale, HI = Hyperactivity-impulsivity subscale, ARS = ADHD Rating Scale, ASRS = ADHD Self-Report Scale, ADHD-RS = Attention-Deficit Hyperactivity Disorder Rating Scale, ASRS-V = Adult ADHD Self-Report Screening Scale for DSM-5, CADS = ADHD Rating Scale/DSM-IV Scale, SNAP-IV = Swanson Nolan and Pelham-IV rating scale, CTD = Chronic Tic Disorder, PUTS = Premonitory Urge for Tics Scale, TS = Tourette Syndrome

Table 7: Associations with Mood/Anxiety Symptoms/Disorders

| **Study** | **Sample Size/Demographics** | **Findings** |
| --- | --- | --- |
| Rozenman et al (2015) (14) | 124 children and adolescents with CTDs | No significant group difference in PUTS scores between those with and without a comorbid anxiety disorder (statistical values not reported in publication)  Participants with elevated anxiety symptoms (SCARED-C ≥ 22 and/or CY-BOCS ≥ 14) had significantly higher PUTS scores (t = -3.54, p = 0.001) than those without elevated anxiety symptoms.  SCARED-C panic/somatic subscale score was a significant predictor of PUTS scores (β = 0.37, p < 0.01) in a stepwise multiple linear regression with age and YGTSS impairment as covariates. |
| McGuire et al (2016) (43) | 75 children with any tic disorder | Significant correlation between PUTS scores and SCARED-C (Spearman’s r = 0.48, p < 0.001) and SCARED-P scores (Spearman’s r = 0.26, p < 0.05). |
| Eddy and Cavanna (2014) (26) | 100 adolescents and adults with CTDs | Significant correlation between PUTS scores and HADS anxiety and depression subscale scores (Pearson’s r = 0.351, p = 0.001, Pearson’s r = 0.254, p = 0.014 respectively)  Correlation with depression subscale of HADS did not survive correction for multiple comparisons. |
| Narapareddy et al (2022) (32) | 48 adults with CTDs | Significant correlation between GAD-7 and PUTS scores (Spearman’s r = 0.40, p-value not reported but below the significance threshold established by Benjamini et al’s false discovery rate-controlling procedure)  No significant correlation between PHQ-9 and PUTS scores (Spearman’s r = -0.04, p-value not reported but above significance threshold established by Benjamini et al’s false discovery rate-controlling procedure) |
| Crossley and Cavanna (2013) (51) | 72 adolescents and adults with TS | Significant correlation of PUTS scores with HADS total (Spearman’s r = 0.28, p = 0.019) and anxiety subscale scores (Spearman’s r = 0.33, p = 0.005). No significant correlation with HADS depression subscale scores (Spearman’s r = 0.18, p = 0.136). |
| Brandt et al (2023) (52) | 291 adults with CTDs | Comorbid depression was significantly associated with presence of premonitory urges (B = 2.07, p = 0.008) in a binary logistic regression model that included age, gender, and other psychiatric comorbidities as covariates. |
| Gulisano et al (2015) (4) | 95 children and adolescents with TS | No significant correlation between PUTS scores and MASC or CDI scores at follow-up (Spearman’s r = 0.158 (p = 0.13) and 0.188 (p = 0.19), respectively). |
| Steinberg et al (2013) (25) | 56 children and adolescents with CTDs | No significant correlation between SCARED or CDI scores and PUTS scores (Pearson’s r = 0.07 and 0.10, respectively; p > 0.01 for both). |
| Isaacs et al (2021) (31) | 52 adults with CTDs | No significant correlation between PUTS scores and GAD-7 (Spearman’s r = 0.29, p > 0.001) or PHQ-9 scores (Spearman’s r = 0.25, p > 0.001) |
| Steinberg et al (2010) (3) | 40 children with CTDs | Significant correlation between SCARED and PUTS scores (r = 0.34, p < 0.05). No significant correlation of PUTS with CDI scores (r = 0.24, p = 0.15).  After dividing the sample into younger (10 years and younger) and older (older than 10 years) subgroups, the only significant correlation between PUTS scores and either scale was a significant correlation between CDI and PUTS scores in the older group (r(22) = 0.47, p < 0.05) (correlation type unclear from publication) |
| Woods et al (2005) (2) | 42 children and adolescents with CTDs | Significant correlation between PUTS and CBCL withdrawal and anxiety/depression subscale scores (Pearson’s r = 0.38 and 0.33, respectively, p < 0.05 for both).  When dividing the sample into youth 10 years and younger and older than 10 years, these correlations only remained significant in the older group (r = 0.59, p < 0.01 and r = 0.55, p < 0.01, respectively) |

HADS = Hospital Anxiety and Depression Scale, PHQ-9 = Patient Health Questionnaire-9, MASC = Multidimensional Anxiety Scale for Children, CDI = Children’s Depression Inventory, CBCL = Child Behavior Checklist, SCARED-C = Screen for Child Anxiety Related Emotional Disorders – Child Version, SCARED-P = Screen for Child Anxiety Related Emotional Disorders – Parent Report, CY-BOCS = Children’s Yale-Brown Obsessive-Compulsive Scale, YGTSS = Yale Global Tic Severity Scale, GAD-7 = Generalized Anxiety Disorder Scale-7, CTD = Chronic Tic Disorder, PUTS = Premonitory Urge for Tics Scale, TS = Tourette Syndrome

Table 8: Associations with Behavioral, Conduct, and Emotional Problems

| **Study** | **Sample Size/Demographics** | **Findings** |
| --- | --- | --- |
| Woods et al (2005) (2) | 42 children and adolescents with CTDs | Significant positive Pearson’s correlations between PUTS scores and social problems (r = 0.43, p < 0.05) and aggressive behavior (r = 0.43, p < 0.05) subscale scores of CBCL in youth older than 10 years of age  Pearson’s correlations between PUTS scores and social problems (r = -0.29, p > 0.05) and aggressive behavior (r = 0.12, p > 0.05) subscale scores of CBCL not significant youth 10 years of age and younger |
| Gulisano et al (2015) (4) | 95 children and adolescents with TS | No significant correlation found between PUTS and CBCL scores (Spearman's r = 0.17, p = 0.14). |
| Silvestri et al (2019) (54) | 45 children with CTDs | Significant positive correlation between PUTS score and difficulty identifying feelings score of AQC (Pearson's r = 0.41, p = 0.005).  The DIF score of the AQC was also a significant predictor of PUTS scores in a multiple linear regression (β = 0.39, p = 0.014) that included gender of the child, mother’s education level, parenting stress index total score, and the DIF score of the Toronto Alexithymia scale as other covariates |
| Ramsey et al (2021) (27) | 75 children with any tic disorder | Significant positive correlation PUTS and DTS (r = 0.41, p < 0.001) such that higher PUTS scores were associated with lower levels of distress tolerance (correlation type unclear from publication) |
| Ramsey et al (2022) (38) | 80 adults with TS or chronic motor tic disorder | Significant positive correlation between PUTS and DTS (r = 0.39, p = 0.001) such that higher PUTS scores were associated with lower levels of distress tolerance (correlation type unclear from publication) |
| McGuire et al (2016) (43) | 75 children with any tic disorder | Significant negative correlation between PUTS and DTS (Spearman's r = -0.40, p < 0.001) *Note, in this case higher DTS scores were said to correspond to greater distress tolerance.  No significant correlations between any of the three I-PUTS dimensions and DTS scores (Spearman’s r = 0.03, 0.04, 0.06) |
| Hagstrøm et al (2021) (55) | 65 children with TS | Neither PUTS score nor the presence of premonitory urge (defined as “a score of 3 or 4 on the PUTS in any of the first six questions”) was associated with emotional regulation scores as assessed by the “The Tangram Emotion Coding Manual” (Pearson’s r = 0.111, p = 0.377, point-biserial r = -0.064, p = 0.612) |
| Openneer et al (2020) (7) | 656 youth with CTDs | No correlation found between externalizing symptom severity (measured by hyperactivity and conduct subscales of SDQ) and PUTS scores (Pearson's r = 0.047, p > 0.05).  After dividing the sample into children 7 years and younger, 8-10-years-old, and older than 10 years, this correlation became significant only in 8-10-years-old group (n = 253, Pearson’s r = 0.149, p < 0.05)  Small positive correlation found between internalizing symptom severity (measured by emotional problems and peer problems subscales of SDQ) and PUTS scores (Pearson's r = 0.084, p < 0.05).  After dividing the sample into children 7 years and younger, 8-10-years-old, and older than 10 years, this correlation only remained significant in the 8-10-years-old group (n = 253, Pearson’s r = 0.177, p < 0.001) |

AQC = Alexithymia Questionnaire for Children, DIF = Difficult Identifying Feelings, DTS = Distress Tolerance Scale, SDQ = Strengths and Difficulties Questionnaire, CBCL = Child Behavior Checklist, CTD = Chronic Tic Disorder, PUTS = Premonitory Urge for Tics Scale, I-PUTS = Individualized Premonitory Urge for Tics Scale, TS = Tourette Syndrome

Table 9: Associations with Quality of Life and Global Functioning

| **Study** | **Sample Size/Demographics** | **Findings** |
| --- | --- | --- |
| Ramsey et al (2021) (27) | 75 children and adolescents with any tic disorder | Significant correlations between PUTS scores and CSDS-P (r = 0.32, p = 0.006) and CSDS-C scores (r = 0.35, p = 0.002) (correlation type unclear from publication)  Additionally, the authors used structural equation modeling to examine the theoretical construct of urge intolerance, a construct derived from items on the PUTS and DTS. Higher levels of urge intolerance predicted both parent- and child- rated disability (β = 2.65, p = 0.008 and β = 4.21, p < 0.001 respectively) |
| Crossley and Cavanna (2013) (51) | 72 adolescents and adults with TS | Significant correlation between PUTS and GTS-QoL (higher scores indicate poorer health-related QoL) scores (Spearman's r = 0.41, p < 0.001) |
| Eddy and Cavanna (2014) (26) | 100 adolescents and adults with TS | Significant correlation between PUTS and GTS-QoL scores (Pearson's r = 0.367, p < 0.001) |
| Langelage et al (2022) (53) | 25 children and adolescents with TS | GTS-QoL scores correlated with mean urge intensity reported during five minutes of a live urge monitor assessment (Pearson's r = 0.50, p = 0.036) |
| Cavanna et al (2012) (56) | 46 individuals with TS (Baseline: mean age = 10.8, Follow-up: mean age = 23.9) | Presence of premonitory urges in childhood was associated with higher GTS-QoL scores (i.e., poorer health-related QoL) in adulthood (t(44) = 2.14, p = 0.038)  Additionally, the following independent variables (all at baseline) were entered into a stepwise multiple linear regression model predicting GTS-QoL total scores in adulthood: family history of tics, presence of premonitory urges, coprolalia, echopraxia, echolalia, and self-injurious behaviors, and diagnoses of OCD and ADHD. Presence of premonitory urges at baseline significantly predicted GTS-QoL scores in adulthood (β = 0.34, p = 0.008)  Presence of premonitory urges at baseline also predicted GTS-QoL obsessive-compulsive domain scores (β = 0.35, p = 0.008) and physical domain scores (β = 0.35, p = 0.008) in similar multiple linear regression models |
| Capriotti et al (2013) (39) | 118 youth with CTDs | Positive correlation between PUTS scores and 12 of 15 tic-related impact items (assessed by questionnaire including both Likert-type items and dichotomous (yes/no) questions) - private leisure, social life, friend relationships, family relationships, home chores, classes, study, school general, feel abnormal, picked on, gossip, and excluded |
| Kano et al (2015) (34) | 41 children and adults with TS | PUTS scores negatively correlated with GAF scores (higher scores indicate greater global functioning) (Pearson’s r = -0.38, p = 0.015) |
| Kano et al (2020) (41) | 20 adolescents and adults with TS (participants had also been assessed an average of 4.0 years prior) | Previous PUTS scores were not significantly correlated with current GAF scores (Pearson’s r = -0.327, p = 0.186), but change in PUTS scores between baseline and follow-up was correlated with change in GAF scores between baseline and follow-up (Pearson’s r = 0.518, p = 0.023) |
| Brandt et al (2023) (52) | 291 adults with CTDs | Higher urge intensity (measured on 1–11-point scale) correlated with lower quality of life (Pearson’s r = 0.28, p < 0.001) (Health-related QoL assessed with GTS-QoL, though it is unclear precisely which components of the GTS-QoL were used based on the information provided) |
| Brandt et al (2023) (40) | 111 adults with TS | Significant correlation between PUTS and GTS-QoL scores (Spearman’s r = 0.44, p < 0.001) |
| Isaacs et al (2021) (31) | 52 adults with CTDs | PUTS scores did not significantly correlate with GTS-QoL scores after correction for multiple comparisons (Spearman's r = 0.36, p > 0.001) |
| Narapareddy et al (2022) (32) | 48 adults with CTDs | GTS-QoL scores did not significantly correlate with PUTS scores after correction for multiple comparisons (Spearman's r = 0.22, p-value not reported but above the significance threshold established by Benjamini et al’s false discovery rate-controlling procedure) |
| Ganos et al (2012) (33) | 15 adults with TS | No significant correlation between PUTS and GTS-QoL scores (Pearson’s r = 0.322, p = 0.242) |

CSDS-P = Child Sheehan Disability Inventory – Parent Rated, CSDS-C = Child Sheehan Disability Inventory – Child Rated, GTS-QoL = Gilles de la Tourette syndrome-quality of life scale, GAF = Global Assessment of Functioning, DTS = Distress Tolerance Scale, OCD = obsessive-compulsive disorder, ADHD = attention-deficit/hyperactivity disorder, CTD = Chronic Tic Disorder, PUTS = Premonitory Urge for Tics Scale, TS = Tourette Syndrome

Table 10: Premonitory Urge Response to Behavioral Interventions

| **Study** | **Sample Size/Demographics** | **Type of Intervention** | **Findings** |
| --- | --- | --- | --- |
| Kang et al (2022) (57) | 30 children with CTDs | CBIT (eight weekly sessions, 90 minutes each) vs supportive psychotherapy | No significant between-group difference in PUTS score change from pre- to post-intervention (CBIT group n = 18; supportive psychotherapy group n = 12; Mann-Whitney U = 88.50, p = 0.415). Pre- to post-intervention change in PUTS scores was also not significant for either group. |
| Houghton et al (2017) (58) | 126 children and 122 adults with CTDs | CBIT vs PST (eight sessions over 10 weeks) | A 2x2x3 (treatment condition × response status × time) repeated measures ANOVA was conducted to assess factors that contributed to the change in PUTS scores over the trial.  In the pediatric trial, there were no significant main effects (time: F(2, 204) = 1.59, p = 0.21, treatment condition: F(1, 102) = 0.52, p = 0.47, response status: , F(1, 102) = 0.69, p = 0.41) or interactions.  In the adult trial, there were no significant main effects of treatment condition or response status (treatment condition: F(1, 95) = 0.003, p = 0.96, response status: F(1, 95) = 0.30, p = 0.59), but there was a significant main effect of time (F(2, 190) = 4.55, p = 0.012), such that PUTS scores decreased over time across all participants.  A bootstrapping regression technique investigated whether reduction in premonitory urges mediated the relationship between treatment condition and reduction in YGTSS total tic score: reductions in premonitory urge severity did not mediate the relationship between treatment and outcome (child trial: effect = 0.003, adult trial: effect = -0.03). |
| Deckersbach et al (2014) (59) | eight adults with CTDs | CBIT for all participants (10 weeks) | Mean PUTS scores at baseline were 26.00 and post-treatment were 25.57 |
| Nonaka et al (2015) (60) | six participants aged 9-20 with TS | CBIT for all participants (10 weeks) | Mean PUTS scores did not significantly change, while paired sample t-tests showed that YGTSS-TTS (t = 2.8, p = 0.04) and YGTSS Impairment Scores (t = 3.8, p = 0.01) significantly improved |
| Verdellen et al (2008) (61) | 18 children and adults with TS | two training sessions (two hours each) followed by 10 weekly, two hour ERP treatment sessions | Across participants SUD-score tended to decrease both within sessions (p < 0.001) and across the 10 sessions (p < 0.001). Also, greater tic frequency (as counted by the therapist) during a session was associated with a lesser reduction in SUD-score within that session. The reduction in SUD-score within a session was found to be independent of session number (p = 0.48). |
| Van de Griendt et al (2023) (62) | 29 children and adults with CTDs | two training sessions and 10 weekly, one hour sessions of ERP | Urge ratings tended to increase during each session, with a repeated measures analysis showing an increase in urge intensity within sessions (F (1.49, 41.63) = 5.81, p < 0.05). A piecewise model showed that this increase tended to occur during the first 15 minutes of each session, followed by stability in the remainder of each session |
| Heijerman-Holtgrefe et al (2021) (63) | 14 children with TS (13 completed the study) | three consecutive days of group-based ERP sessions, followed by one more session a week later (each session was 135 minutes) | While YGTSS-TTS scores significantly reduced, mean PUTS scores declined by only 3% (No further statistical data was provided regarding change in PUTS) |
| Nissen et al (2019) (64) | 54 children and adolescents with CTDs (n = 27 in each group) | HRT/ERP administered in groups of four vs. HRT/ERP administered individually - each treatment group consisted of eight sessions plus one booster session | No significant difference in PUTS scores between baseline and the eighth session in both group and individual administration of HRT/ERP (values not reported)  No association between the change in PUTS scores from baseline to post treatment and the change in either YGTSS-TTS or YGTSS Impairment from baseline to post-treatment (p = 0.24 and p = 0.56, respectively) |
| Sukhodolsky et al (2017) (65) | 248 children and adults with CTDs | CBIT vs. PST (eight sessions over 10 weeks) | A mixed-model repeated-measures analyses showed that higher PUTS scores at baseline significantly predicted lesser reduction of YGTSS-TTS scores across both treatment groups (F_1,225_ = 8.13, p = 0.005). PUTS scores did not moderate treatment response |

CBIT = Comprehensive behavioral intervention for tics, ERP = Exposure and response prevention, HRT = Habit reversal training, PST = Psychoeducation and supportive therapy, SUD-score = subjective units of distress scale score (higher scores indicate more distressing premonitory urges), YGTSS = Yale Global Tic Severity Scale, TTS = Total Tic Score, CTD = Chronic Tic Disorder, PUTS = Premonitory Urge for Tics Scale, TS = Tourette Syndrome

Table 11: Premonitory Urge Response to Pharmacotherapy

| **Study** | **Sample Size/Demographics** | **Type of Intervention** | **Findings** |
| --- | --- | --- | --- |
| Gerasch et al (2016) (23) | 44 adults with TS (18 chose treatment – open label) | Aripiprazole (Abilify) - dose began at 2.5 mg/day and was increased every three days to a maximum of 30 mg/day (mean final dose was 12.2 mg). The treatment lasted four to six weeks  Prior to treatment participants completed a four week washout period if they were using psychoactive substances | A Mann-Whitney U test for paired samples showed no significant difference in PUTS scores between baseline and follow up for the treatment group (mean difference = 0.1, p = 0.917) but there was a significant reduction in YGTSS-TTS scores between baseline and follow up for the treatment group (mean difference = -3.5, p = 0.027) |
| Gilbert et al (2014) (66) | 18 (15 completed) adults with TS – open label | Ecopipam – eight weeks  - 50 mg each night for two weeks, then 100 mg each night for six weeks | PUTS scores did not significantly change from baseline (mean = 25.0) to study completion (mean = 25.4), but YGTSS-TTS (p = 0.0008) and YGTSS impairment (p = 0.0161) did decrease from baseline to study completion |
| Jankovic et al (2010) (67) | 29 (20 completed) children and adults with TS | Randomized, double blind, placebo-controlled trial of topiramate – Started on 25 mg/day of topiramate, with doses increasing over the course of six weeks to a maximum of 200 mg/day, followed by a four week maintenance phase and subsequent 12-day taper phase | YGTSS-TTS scores significantly improved from baseline to day 70. Premonitory urge Clinical Global Impression also improved (values not reported) |
| McGuire et al (2020) (68) | 20 children with CTDs | Randomized trial of one session of HRT combined with 50 mg D-cycloserine taken one hour before vs. one session of HRT with placebo | Conducted a fixed effects repeated measures analysis with a treatment group factor and baseline tic severity, co-occurring ADHD and alpha-agonist medication as covariates. There was a significant interaction between treatment group and time (p = 0.02) for I-PUTS frequency, such that I-PUTS frequency declined more in the treatment group. There was no significant interaction between treatment group and time (p = 0.13) for I-PUTS intensity |
| Jankovic (1994) (69) | 10 patients (aged 13-53) with TS | BTX - 30 U to 300 U (depending on the site of injection) | Patients were assessed two to four months following the injection, during which they rated the effect of the injection of their premonitory urges on a 0-3 scale (higher values indicate greater response, more effective treatment). Of the seven patients that experienced premonitory urges at baseline, all seven responded with a two or three on the 3-point scale |
| Kwak et al (2000) (70) | 35 children and adults (34 with TS, one meeting all diagnostic criteria for TS other than age of tic onset) | BTX - varying number of injections over an average of just under two years (mean number of visits per participant = 3.3) | Participants reported their percent improvement in premonitory urges following BTX injection, and 84% of the 25 patients who experienced premonitory urges at baseline “derived mark relief of these symptoms”. These participants mean reported percentage improvement in premonitory urges was 70.6%, with three participants reporting “complete resolution of premonitory discomfort” |
| Marras et al (2001) (71) | 18 individuals (IQR = 15-55) with TS or chronic motor tic disorder | Double blind, randomized, placebo-controlled crossover trial of BTX to muscles involved in target tics (each phase was one injection, follow up assessments done at 2 weeks post injection) | Urge (urge to perform the tic) ratings decreased significantly more in the BTX phase compared to placebo phase (p = 0.02), while there was no significant difference between changes in premonitory sensation (sensation associated with a tic) ratings (p = 0.47) between the phases. (unclear what the operational difference in these constructs is) |
| Kwak et al (2003) (72) | 50 children and adults with TS (12 participants reported having received BTX injections) | BTX | 12 participants reported having received BTX injections for motor tics in the past and 8 (67%) of these participants reported that these injections reduced their premonitory urges.  Of note, 13 of 19 (68%) participants reported that medication taken for their tics or comorbid OCD reduced their premonitory urges as well. |
| Rath et al (2010) (73) | 15 adults with CTDs | BTX-A - every three to four months with a varying total number of injections (median number of treatments per tic = 6) | Of the eight patients that experienced premonitory urges, all had their premonitory urges reduced following BTX-A treatment, and two patients reported that their premonitory urges had completely gone away following their first two treatments. |
| Porta et al (2004) (74) | 28 children and adults with TS and two adults that met all TS criteria except age of tic onset | BTX-A injected into vocal cords - number of treatments (mean = 1.9) and interval between treatments (mean = 4.2 months) varied | After treatment, only 20% of patients reported experiencing premonitory urges compared to 53% prior to treatment. |
| Di Lazzaro et al (2020) (75) | three adults with tic disorders | BTX | Patients “felt frustrated for still experiencing the need to tic in the treated areas, without being able to do so due to BoNT-induced muscle weakness" |
| Müller-Vahl et al (2021) (76) | 44 adults with TS | Lu AG06466 (monoacylglycerol lipase inhibitor) - eight week, double blind randomized, placebo-controlled trial – active group treatment: “10 mg for the first 3 days, 20 mg on days 4–28, 30 mg on days 29–35, before the target dose of 40 mg was reached for 21 days until day 56” | No significant difference in PUTS scores between groups at four (p = 0.50) or eight weeks (p = 0.67). |
| Müller-Vahl et al (2022) (77) | 19 adults with TS | Lu AG06466 - single dose, double blind placebo-controlled crossover trial | PUTS scores decreased significantly more in the treatment condition compared to the placebo condition at 4 and 12 hours post dose (p < 0.05), but not at 8 hours post dose |
| Müller-Vahl et al (1998) (78) | 64 adolescents and adults with TS | Cannabis – interview of TS patients’ experiences using marijuana | 17 reported previously using cannabis, and four of these patients reported "reduction in pre-urges” |
| Anis et al (2022) (79) | 18 adults with TS (15 completed the study) | Cannabis - open label study of daily cannabis (varying doses and methods of administration) | PUTS scores significantly decreased after 4 and 12 weeks (p = 0.043 at both timepoints)  Many of the subjects reported side effects including dry mouth (67%), fatigue (53%), and sedation and dizziness (47%) |
| Abi-Jaoude et al (2022) (80) | 12 adults with TS (9 completed the study) | three treatments (all vaporized medical cannabis): ∆9 -tetrahydrocannabinol (THC), Cannabidiol (CBD), THC/CBD combination - double blind, randomized placebo-controlled crossover study - each participant received each product once, with each condition separated by two weeks | The THC and THC/CBD products significant reduced PUTS scores relative to placebo (THC: at 30 min, one hour, two hours, and three hours after administration (p < 0.001), THC/CBD: at one hour (p < 0.05), two hours and three hours (p < 0.001) after administration) |
| Silver et al (1999) (81) | one 58-year-old male with “Tourette symptoms” | 7 mg transdermal nicotine patch | Disappearance of premonitory urges and tics within three hours, symptoms returned seven days later, reported nausea as a side effect |
| Szejko et al (2020) (82) | one 46-year-old female with TS and Guillain-Barré-like immune-mediated neuropathy | Intravenous immunoglobulin (IVIg) - five days of 30g per day | Temporary reduction in premonitory urges after five days of IVIg treatment |
| Szejko et al (2019) (83) | One 12-year-old male with TS | 0.15g of vaporized cannabis (33mg of THC) | PUTS score decreased from 23 at baseline to 15 at 30 minutes post inhalation of cannabis |

BTX = Botulinum toxin, IQR = inter quartile range, YGTSS = Yale Global Tic Severity Scale, TTS = Total Tic Score, CTD = Chronic Tic Disorder, PUTS = Premonitory Urge for Tics Scale, I-PUTS = Individualized Premonitory Urge for Tics Scale, TS = Tourette Syndrome, HRT = Habit reversal training, ADHD = attention-deficit/hyperactivity disorder, OCD = obsessive-compulsive disorder, g = grams, mg = milligrams, THC = tetrahydrocannabinol, CBD = Cannabidiol, IVIg = intravenous immunoglobulins

Table 12: Neural Correlates of Premonitory Urge

| **Study** | **Sample Size/Demographics** | **Neuroimaging Technique** | **Findings** |
| --- | --- | --- | --- |
| Bhikram et al (2021) (84) | 40 adults with TS | fMRI (participants completed a blink suppression task that included four free to blink and four blink suppression conditions, and during these conditions participants were shown either angry or neutral facial expressions) | No significant associations between PUTS scores and brain activity in free to blink vs. blink suppression conditions across emotional conditions  PUTS scores were significantly positively associated with the interaction between emotional condition and blink condition on activity in the thalamus (z = 3.97, p < 0.001), hippocampus (z = 4.17, p < 0.001), mid temporal gyrus (z = 4.79, p < 0.001), precuneus (z = 4.17, p < 0.001), midcingulate (z = 4.70, p < 0.001), and the tail of the caudate (z = 4.33, p < 0.001) such that activity was greater in the angry face conditions than the neutral face conditions |
| Eddy et al (2016) (85) | 25 adults with TS | fMRI (while participants completed a theory of mind task) | Associations between PUTS scores and brain activity during theory of mind tasks in right temporal-parietal junction (TPJ) (F = 13.02, p = 0.002) and left (F = 6.46, p = 0.021) and right (F = 10.30, p = 0.005) amygdalae but not left TPJ or right posterior cingulate. |
| Eddy et al (2017) (86) | 25 adults with TS (same sample as Eddy et al 2016) | fMRI (while participants completed a “reading the eyes” task) | PUTS scores were associated with greater activity in the right (t = 2.91, p = 0.009) and left TPJ (t = 2.23, p = 0.037) during the task, but no significant association was found between PUTS scores and left or right amygdalae activity during the task |
| Deckersbach et al (2014) (59) | 8 adults with TS | fMRI (while participants completed a visuospatial priming task that “assesses the effects of an inhibitory or faciliatory ‘prime’ on the reactions to a subsequently presented probe”) – PUTS and fMRI task completed before and after 10 weeks of CBIT | Prior to CBIT, task-related activation in the superior temporal gyrus was negatively correlated with PUTS scores (Pearson’s r = -0.97, p < 0.001). The change in PUTS score from pre- to post- CBIT did not significantly correlate with the change in task-related activation from pre- to post- CBIT. Pre-CBIT PUTS scores were not significantly correlated with change in task-related activation in five *a priori* regions of interest (Brodmann's area 11, 44, 47, caudate and putamen) |
| Rae et al (2020) (87) | 23 adults with TS | fMRI (while participants completed a “Go/NoGo” task) | No correlations of PUTS scores with task effects for either Choose-Go (“volitional action”) or Choose-NoGo (“intentional inhibition”) conditions. However, during trials in which participants “chose-go,” PUTS scores were positively associated with functional connectivity of the pre-SMA with the following regions (p < 0.05 FDR cluster corrected; cluster-forming threshold: p<0.001): caudate nucleus, globus pallidus and thalamus. There were no associations between PUTS score and IFG functional connectivity |
| Rae et al (2023) (88) | 23 adults with TS (same sample as Rae et al 2020) | fMRI (while participants completed a “Go/NoGo” task) | Greater representational similarity in the caudate nucleus between "go" and "nogo" conditions positively correlated with PUTS scores (r = 0.513, p = 0.012), but this association did not survive correction for multiple comparisons. |
| Tinaz et al (2015) (89) | 13 adults with TS | resting state fMRI | PUTS scores were positively associated with functional connectivity between the right dorsal anterior insula and the right SMA2 (F = 9.49, t = 3.08, p = 0.012) and left SMA1 (F = 5.78, t = 2.40, p = 0.037) in multiple regression analysis (see publication for MNI coordinates corresponding to “SMA1” and “SMA2”). |
| Rae et al (2018) (90) | 21 adults with TS | fMRI (while undergoing fMRI, participants completed a face perception in which they viewed angry or neutral faces and were instructed to report whether the face was male or female) | Contrasts in both the neutral face and angry face conditions (relative to baseline) were not significantly associated with PUTS scores. However, functional connectivity between the insula and the following regions was positively associated with PUTS scores (p < 0.05 FDR cluster corrected; cluster-forming threshold: p<0.001): SMA, posterior cingulate, precuneus, and fusiform gyrus/cerebellum |
| Bhikram et al (2020) (91) | 40 adults with TS | resting state fMRI | PUTS scores were negatively associated with functional connectivity between the insula and inferior frontal gyrus (t = 4.69, p = 0.029), between the orbitofrontal cortex and pre/post central gyri (t = 5.65, p < 0.001), and between the putamen and inferior frontal gyrus (t = 4.62, p = 0.029). |
| Tinaz et al (2014) (92) | 15 adults with TS (16 enrolled but 1 TS participants’ MRI data were not usable) | 1. resting state fMRI 2. MEG 3. MRI 4. MRS | 1. No significant associations between PUTS scores and sensorimotor cortex functional connectivity. 2. No significant correlations between PUTS scores and baseline beta band power in sensorimotor cortex (r = 0.10, p = 0.74) 3. No significant correlations between PUTS scores and sensorimotor cortical volume (Left SMC: r = -0.22, p = 0.43, Right SMC: r = -0.43, p = 0.10). 4. GABA+ / Creatine ratio in the sensorimotor cortex did not significantly correlate with PUTS scores r = -0.40, p = 0.15).  - (correlation types unclear from publication) |
| Loo et al (2019) (93) | 23 children with CTDs | EEG (while participants completed cued eye-blink task) | Positive correlations of “urge strength” with gamma power in dorsolateral prefrontal cortex (Pearson’s r = 0.42, p < 0.05) and SMA (Pearson’s r = 0.44, p < 0.05), and of “urge strength” with alpha power in the left parietal cortex (Pearson’s r = 0.39, p < 0.05); negative correlations of “urge strength” with alpha power in the posterior cingulate (Pearson’s r = -0.49, p < 0.05).  Note: 1) activations are around the time of cued blinks, 2) unclear which items of PUTS correspond to “urge strength” |
| Niccolai et al (2019) (94) | 11 adults with TS (PUTS data for n = 8) | MEG | Beta power slope of bilateral premotor and motor cortices in time window one (1s to 0.5s before tics) significantly correlated with PUTS item 6 ("I feel like there is energy in my body that needs to get out") (r = 0.928, p = 0.004). Spearman’s correlations between total PUTS scores and beta power slopes in time window one (r = 0.710, p = 0.055) and time window two (0.5s to 0s before tics) (p = 0.564) were not significant. |
| Ganos et al (2014) (95) | 14 adults with TS | fMRI (while participants completed free to tic and tic suppression conditions) | No correlation between PUTS scores and regional homogeneity contrast values between suppression and free to tic conditions in left inferior frontal gyrus (Pearson’s r = -0.091, p = 0.758).  *Note: regional homogeneity “measures the local synchronization of spontaneous fMRI signals… and can therefore be considered a measure of local connectivity” |
| He et al (2022) (96) | 68 children with TS | MRS – right hemisphere | After correcting for multiple comparisons, there were significant negative Pearson’s correlations of SMA GABA+ concentration with I-PUTS frequency (r = -0.39, p = 0.022), I-PUTS intensity (r = -0.42, p = 0.011), and I-PUTS number (r = -0.40, p = 0.02), such that lower levels of SMA GABA+ were associated with greater premonitory urge symptoms. There were no significant associations of GABA+ concentrations in the insula or SMC with I-PUTS scores, nor were there any associations between Glx levels in any of the three brain regions and I-PUTS scores |
| Kanaan et al (2017) (97) | 37 adults with TS | MRS | Significant negative association between thalamic glutamate concentration and PUTS scores (r = -0.47, p = 0.017), after adjusting for age and sex |
| Draganski et al (2010) (98) | 34 adults with TS | MRI (VBM approach) | Significant positive associations between PUTS scores and both left somatosensory cortex (S1) and right prefrontal cortex (PFC) grey matter volume (z = 4.3, p < 0.05, z = 4.7, p < 0.05 respectively) and cortical thickness (z = 4.7, p < 0.05, z = 4.2, p < 0.05 respectively). Also, the authors identified a positive association between PUTS scores and left dorsal premotor cortex grey matter volume (z = 4.3, p < 0.05) and a negative association between PUTS scores and fractional anisotropy of white matter for portions of the bilateral superior longitudinal fascicles (p < 0.05) - Age, gender and total intracranial volume were controlled for as covariates |
| Draper et al (2016) (36) | 29 individuals aged 8-21 with TS | MRI | No positive associations between regions of grey matter thickness and PUTS scores, but there were significant negative associations between PUTS scores and grey matter thickness in clusters within the following regions: Right Rolandic Operculum, Left Inferior Occipital Gyrus, Left insula, and Left Pre-Central Gyrus  (A multi-step process was used to arrive at significance: “An initial height threshold was set at p < .001, and identified clusters were then corrected for multiple comparisons (p < .05) and an extent threshold of 10 mm 2 was applied.”)  (Table 2 in the cited publication provides cluster sizes of the above regions; magnitude of the effect is not reported) |
| Sigurdsson et al (2018) (99) | 28 individuals aged 8-21 with TS | DTI (analyzed using graph theory) | No association between any measure of diffusion and PUTS scores (p > 0.05). Significant positive associations between PUTS scores and local efficiency (“efficacy of information transfer”) in the right posterior insula (thresholds: 21-18%, β = 0.55, p < 0.001) as well as the left anterior insula (thresholds: 16-15%, β = 0.60, p < 0.001). There was also a significant negative association between local efficiency in the left caudate and PUTS scores (thresholds: 27%-24%, 22%-20%, β = -0.55 to -0.57, p < 0.001) - age, IQ, and total intracranial volume were controlled for as covariates |
| Sigurdsson et al (2020) (100) | 28 adolescents with TS | MRI (VBM approach) - focusing on regions of interest within the cerebellum | PUTS scores were positively correlated with grey matter volume in a cluster in the right cerebellar lobule VI (t = 3.88, p < 0.001)  (correlation type unclear from publication) |
| Sigurdsson et al (2020) (101) | 16 children and adults with TS | Neuronavigated transcranial magnetic stimulation (nTMS) and EMG recording to map the representation of different muscles in the sensorimotor cortex | Significant negative correlations (controlling for age and sex) between the PUTS and the Euclidean distance between the first dorsal interosseous (FDI, hand) and the orbicularis oris (lips) (Pearson’s r = -0.67, p = 0.012), the Euclidean distance between the FDI and masseter (jaw) (Pearson’s r = -0.78, p = 0.002), and the Euclidean distance between the FDI and orbicularis oculi (eyes) (Pearson’s r = -0.69, p = 0.005). The area of FDI cortical representation also was positively correlated with PUTS scores (Pearson’s r = 0.60, p = 0.01) |
| Jackson et al (2020) (29) | 28 children and adults with TS | MRI (VBM approach) | Significant positive correlation between grey matter volume in an anterior-dorsal (Area IG 2) cluster within the right insula and PUTS scores (Pearson’s r = 0.43, p < 0.05). Of note, grey matter volume in a separate region of the right insula was negatively correlated with YGTSS motor tic scores |
| Jackson et al (2021) (102) | 28 children and adults with TS (same sample as Jackson et al 2020) | MRI (VBM approach) | Significant positive correlation between grey matter volume in a cluster within the left hemisphere mid-cingulate and PUTS scores (r = 0.44, p < 0.05). Of note, grey matter volume in two other clusters in the left hemisphere cingulate cortex were positively correlated with YGTSS motor tic scores (correlation type unclear from publication) |
| Brandt et al (2014) (42) | 14 adults with TS and 15 age-matched controls | PAS - “Peripheral, electrical stimulation at the wrist and central, TMS stimulation over M1 are repeatedly combined in such a way that both stimuli arrive in the cortex simultaneously, which should result in a transient strengthening of the synapses involved” – Measured MEPs following procedure | Positive correlation between PUTS scores and plasticity (MEP change) following PAS procedure (data from n = 12 with TS, r = 0.82, p = 0.001). Interestingly, although greater premonitory urge severity was associated with a greater LTP-like response, MEP change (plasticity) was lower in those with TS compared to controls  Resting motor threshold correlated with PUTS scores, but not significantly (data from n = 12 with TS, r = 0.53, p = 0.08)  (correlation types unclear from publication) |
| Larsh et al (2022) (44) | 17 children with TS | TMS / EMG | I-PUTS intensity was associated with TMS-evoked cortical excitability (CE) (p < 0.001) and long-interval cortical inhibition (LICI) (p < 0.001) in the left and right motor cortex (M1), such that those with lower TMS-evoked CE and LICI had higher I-PUTS intensity scores  Interestingly, those with lower CE and LICI had lower YGTSS-TTS scores |
| Batschelett et al (2023) (45) | 30 children with TS | TMS / EMG | No significant associations between M1 physiology measures (including short-interval cortical inhibition and intracortical facilitation) and I-PUTS scale scores (all p > 0.5). |
| Hampson et al (2009) (103) | 16 adults with CTDs and matched controls (same gender, handedness, and sex and of similar age) | fMRI (CTD participants were free to tic and matched controls were instructed to imitate the tics of the CTD participant to which they were matched) | Significant group by time interaction for SMA activity around action (p = 0.004, using data from the 14 CTD participants known to experience premonitory urges and their matched controls) such that SMA activity was greater before and after tics in CTD participants than before and after intentional movements in control participants |
| Bohlhalter et al (2006) (104) | 10 adults with TS | fMRI | See Table 2 in publication for areas of statistically significant (p < 0.05, corrected for multiple comparisons) elevations in bold signal two seconds prior to tics. The following regions were also noted to have had stronger fMRI activations two seconds prior to tics than at tic onset: bilateral insula, bilateral posterior putamen, bilateral anterior cingulate cortex, bilateral SMA and bilateral parietal operculum. |
| Duggal et al (2002) (105) | three participants with CTDs (aged 16-51) – all endorsed experiencing premonitory urges | EEG | Bereitschaftspotential (BP, an EEG potential that occurs prior to intentional movements) observed prior to tics closer to tic onset than BP prior to voluntary movement. |
| Morera Maiquez et al (2022) (106) | 16 individuals aged 12-23 with TS | EEG | No desynchronization of mu or beta band power over sensorimotor cortex prior to or at tic onset, unlike during voluntary movements. |
| Zapparoli et al (2024) (107) | 23 individuals with TS | fMRI - included conditions in which they imagined a situation that promotes tic occurrence (“urge phase”) and a condition in which they imagined acting out a tic. Following each phase, participants rated distress or relief experienced during the phase | Distress ratings during the urge phase were positively associated with a network of brain activation including “the superior medial prefrontal cortex and SMA, the middle and inferior frontal gyri, the middle cingulum, bilaterally, the left precentral and postcentral gyri, the left temporo-occipital region, the right fusiform gyrus.” During the urge phase, there were significant clusters of activation in “the right caudate nucleus and nucleus accumbens, the putamen, bilaterally, and the right cerebellum.” (Table 2 in the cited publication provides magnitudes and p-values for each of the above regions.)  Left putamen activity was significantly associated with distress ratings during the “urge phase” and relief ratings during the tic imagination phase |

Glx = glutamate + glutamine, MRI = Magnetic resonance imaging, MRS = Magnetic Resonance spectroscopy, fMRI = Functional magnetic resonance imaging, EEG = Electroencephalography, MEG = Magnetoencephalography, EMG = Electromyography, VBM = voxel-based morphometry, DTI = Diffusion tensor imaging, GABA+ = GABA + macromolecules, SMA = supplementary motor area, TMS = transcranial magnetic stimulation, M1 = Primary motor cortex, S1 = primary sensory cortex, TPJ = temporal-parietal junction, PAS = paired associative stimulus, MEP = Motor Evoked Potential, SMC = sensorimotor cortex, MNI = Montreal Neurological Institute, CE = cortical excitability, LICI = long-interval cortical inhibition, CBIT = Comprehensive behavioral intervention for tics, YGTSS = Yale Global Tic Severity Scale, TTS = Total Tic Score, CTD = Chronic Tic Disorder, PUTS = Premonitory Urge for Tics Scale, I-PUTS = Individualized Premonitory Urge for Tics Scale, TS = Tourette Syndrome

**REFERENCES**

1. Covidence systematic review software.

2. Woods DW, Piacentini J, Himle MB, Chang S. Premonitory Urge for Tics Scale (PUTS): initial psychometric results and examination of the premonitory urge phenomenon in youths with Tic disorders. *J Dev Behav Pediatr* (2005) 26:397–403. doi: 10.1097/00004703-200512000-00001

3. Steinberg T, Shmuel Baruch S, Harush A, Dar R, Woods D, Piacentini J, Apter A. Tic disorders and the premonitory urge. *J Neural Transm (Vienna)* (2010) 117:277–284. doi: 10.1007/s00702-009-0353-3

4. Gulisano M, Calì P, Palermo F, Robertson M, Rizzo R. Premonitory Urges in Patients with Gilles de la Tourette Syndrome: An Italian Translation and a 7-Year Follow-up. *J Child Adolesc Psychopharmacol* (2015) 25:810–816. doi: 10.1089/cap.2014.0154

5. Raines JM, Edwards KR, Sherman MF, Higginson CI, Winnick JB, Navin K, Gettings JM, Conteh F, Bennett SM, Specht MW. Premonitory Urge for Tics Scale (PUTS): replication and extension of psychometric properties in youth with chronic tic disorders (CTDs). *J Neural Transm (Vienna)* (2018) 125:727–734. doi: 10.1007/s00702-017-1818-4

6. Forcadell E, Garcia-Delgar B, Nicolau R, Pérez-Vigil A, Cordovilla C, Lázaro L, Ibáñez L, Mir P, Madruga-Garrido M, Correa-Vela M, et al. Tic disorders and premonitory urges: validation of the Spanish-language version of the Premonitory Urge for Tics Scale in children and adolescents. *Neurologia (Engl Ed)* (2022) doi: 10.1016/j.nrleng.2020.09.005

7. Openneer TJC, Tárnok Z, Bognar E, Benaroya-Milshtein N, Garcia-Delgar B, Morer A, Steinberg T, Hoekstra PJ, Dietrich A. The Premonitory Urge for Tics Scale in a large sample of children and adolescents: psychometric properties in a developmental context. An EMTICS study. *Eur Child Adolesc Psychiatry* (2020) 29:1411–1424. doi: 10.1007/s00787-019-01450-1

8. Li Y, Woods DW, Gu Y, Yu L, Yan J, Wen F, Wang F, Liu J, Cui Y. Psychometric Properties of the Chinese Version of the Premonitory Urge for Tics Scale: A Preliminary Report. *Front Psychol* (2021) 12:573803. doi: 10.3389/fpsyg.2021.573803

9. Baumung L, Müller-Vahl K, Dyke K, Jackson G, Jackson S, Golm D, Münchau A, Brandt V. Developing the Premonitory Urges for Tic Disorders Scale-Revised (PUTS-R). *J Neuropsychol* (2021) 15:129–142. doi: 10.1111/jnp.12216

10. Matsuda N, Nonaka M, Kono T, Fujio M, Nobuyoshi M, Kano Y. Premonitory Awareness Facilitates Tic Suppression: Subscales of the Premonitory Urge for Tics Scale and a New Self-Report Questionnaire for Tic-Associated Sensations. *Front Psychiatry* (2020) 11:592. doi: 10.3389/fpsyt.2020.00592

11. Brandt VC, Beck C, Sajin V, Anders S, Münchau A. Convergent validity of the PUTS. *Front Psychiatry* (2016) 7: doi: 10.3389/fpsyt.2016.00051

12. Edwards KR, Raines JM, Winnick JB, Sherman MF, Higginson CI, Navin K, Conteh F, Ricketts EJ, Specht MW. Sex and psychiatric comorbidity correlates of the premonitory urge for tic scale in youth with persistent tic disorders. *J Neural Transm (Vienna)* (2020) 127:977–985. doi: 10.1007/s00702-020-02151-9

13. Zinner SH, Conelea CA, Glew GM, Woods DW, Budman CL. Peer victimization in youth with Tourette syndrome and other chronic tic disorders. *Child Psychiatry Hum Dev* (2012) 43:124–136. doi: https://dx.doi.org/10.1007/s10578-011-0249-y

14. Rozenman M, Johnson OE, Chang SW, Woods DW, Walkup JT, Wilhelm S, Peterson A, Scahill L, Piacentini J. Relationships between Premonitory Urge and Anxiety in Youth with Chronic Tic Disorders. *Child Health Care* (2015) 44:235–248. doi: 10.1080/02739615.2014.986328

15. Reese HE, Scahill L, Peterson AL, Crowe K, Woods DW, Piacentini J, Walkup JT, Wilhelm S. The premonitory urge to tic: measurement, characteristics, and correlates in older adolescents and adults. *Behav Ther* (2014) 45:177–186. doi: 10.1016/j.beth.2013.09.002

16. Pile V, Lau JYF, Topor M, Hedderly T, Robinson S. Interoceptive Accuracy in Youth with Tic Disorders: Exploring Links with Premonitory Urge, Anxiety and Quality of Life. *J Autism Dev Disord* (2018) 48:3474–3482. doi: 10.1007/s10803-018-3608-8

17. Kyriazi M, Kalyva E, Vargiami E, Krikonis K, Zafeiriou D. Premonitory Urges and Their Link With Tic Severity in Children and Adolescents With Tic Disorders. *Front Psychiatry* (2019) 10:569. doi: 10.3389/fpsyt.2019.00569

18. Kim M, Chung SK, Yang JC, Park JI, Nam SH, Park TW. Development of the Korean Form of the Premonitory Urge for Tics Scale: A Reliability and Validity Study. *Soa Chongsonyon Chongsin Uihak* (2020) 31:146–153. doi: 10.5765/jkacap.200013

19. Crossley E, Seri S, Stern JS, Robertson MM, Cavanna AE. Premonitory urges for tics in adult patients with Tourette syndrome. *Brain Dev* (2014) 36:45–50. doi: 10.1016/j.braindev.2012.12.010

20. Isaacs D, Key AP, Cascio CJ, Conley AC, Walker HC, Wallace MT, Claassen DO. Sensory hypersensitivity severity and association with obsessive-compulsive symptoms in adults with tic disorder. *Neuropsychiatr Dis Treat* (2020) 16:2591–2601. doi: 10.2147/NDT.S274165

21. Schütteler C, Woitecki K, Döpfner M, Gerlach AL. Interoception and Premonitory Urges in Children and Adolescents With Tic Disorders. *Clin Psychol Eur* (2023) 5:e8185-. doi: 10.32872/cpe.8185

22. Sutherland Owens AN, Miguel EC, Swerdlow NR. Sensory gating scales and premonitory urges in Tourette syndrome. *ScientificWorldJournal* (2011) 11:736–741. doi: 10.1100/tsw.2011.57

23. Gerasch S, Kanaan AS, Jakubovski E, Muller-Vahl KR. Aripiprazole improves associated comorbid conditions in addition to tics in adult patients with Gilles de la Tourette Syndrome. *Front Neurosci* (2016) 10:416. doi: https://dx.doi.org/10.3389/fnins.2016.00416

24. Rajagopal S, Cavanna AE. Premonitory urges and repetitive behaviours in adult patients with Tourette syndrome. *Neurol Sci* (2014) 35:969–971. doi: 10.1007/s10072-014-1706-8

25. Steinberg T, Harush A, Barnea M, Dar R, Piacentini J, Woods D, Shmuel-Baruch S, Apter A. Tic-related cognition, sensory phenomena, and anxiety in children and adolescents with Tourette syndrome. *Compr Psychiatry* (2013) 54:462–466. doi: 10.1016/j.comppsych.2012.12.012

26. Eddy CM, Cavanna AE. Premonitory Urges in Adults With Complicated and Uncomplicated Tourette Syndrome. *Behav Modif* (2014) 38:264–275. doi: https://dx.doi.org/10.1177/0145445513504432

27. Ramsey KA, Essoe JK, Storch EA, Lewin AB, Murphy TK, McGuire JF. Urge Intolerance and Impairment Among Youth with Tourette’s and Chronic Tic Disorders. *Child Psychiatry Hum Dev* (2021) 52:761–771. doi: 10.1007/s10578-020-01085-3

28. Barnea M, Benaroya-Milshtein N, Gilboa-Sechtman E, Woods DW, Piacentini J, Fennig S, Apter A, Steinberg T. Subjective versus objective measures of tic severity in Tourette syndrome - The influence of environment. *Psychiatry Res* (2016) 242:204–209. doi: 10.1016/j.psychres.2016.05.047

29. Jackson SR, Loayza J, Crighton M, Sigurdsson HP, Dyke K, Jackson GM. The role of the insula in the generation of motor tics and the experience of the premonitory urge-to-tic in Tourette syndrome. *Cortex* (2020) 126:119–133. doi: 10.1016/j.cortex.2019.12.021

30. Muller-Vahl KR, Riemann L, Bokemeyer S. Tourette patients’ misbelief of a tic rebound is due to overall difficulties in reliable tic rating. *J Psychosom Res* (2014) 76:472–476. doi: https://dx.doi.org/10.1016/j.jpsychores.2014.03.003

31. Isaacs DA, Riordan HR, Claassen DO. Clinical Correlates of Health-Related Quality of Life in Adults With Chronic Tic Disorder. *Front Psychiatry* (2021) 12:619854. doi: https://dx.doi.org/10.3389/fpsyt.2021.619854

32. Narapareddy A, Eckland MR, Riordan HR, Cascio CJ, Isaacs DA. Altered Interoceptive Sensibility in Adults With Chronic Tic Disorder. *Front Psychiatry* (2022) 13: doi: 10.3389/fpsyt.2022.914897

33. Ganos C, Kahl U, Schunke O, Kühn S, Haggard P, Gerloff C, Roessner V, Thomalla G, Münchau A. Are premonitory urges a prerequisite of tic inhibition in Gilles de la Tourette syndrome? *J Neurol Neurosurg Psychiatry* (2012) 83:975–978. doi: 10.1136/jnnp-2012-303033

34. Kano Y, Matsuda N, Nonaka M, Fujio M, Kuwabara H, Kono T. Sensory phenomena related to tics, obsessive-compulsive symptoms, and global functioning in Tourette syndrome. *Compr Psychiatry* (2015) 62:141–146. doi: 10.1016/j.comppsych.2015.07.006

35. Ganos C, Garrido A, Navalpotro-Gómez I, Ricciardi L, Martino D, Edwards MJ, Tsakiris M, Haggard P, Bhatia KP. Premonitory urge to tic in Tourette’s is associated with interoceptive awareness. *Mov Disord* (2015) 30:1198–1202. doi: 10.1002/mds.26228

36. Draper A, Jackson GM, Morgan PS, Jackson SR. Premonitory urges are associated with decreased grey matter thickness within the insula and sensorimotor cortex in young people with Tourette syndrome. *J Neuropsychol* (2016) 10:143–153. doi: 10.1111/jnp.12089

37. Gu Y, Li Y, Cui Y. Correlation between premonitory urges and tic symptoms in a Chinese population with tic disorders. *Pediatr Investig* (2020) 4:86–90. doi: 10.1002/ped4.12189

38. Ramsey KA, De Nadai AS, Espil FM, Ricketts E, Stiede JT, Schild J, Specht MW, Woods DW, Bennet S, Walkup JT, et al. Urge intolerance predicts tic severity and impairment among adults with Tourette syndrome and chronic tic disorders. *Front Psychiatry* (2022) 13:929413. doi: https://dx.doi.org/10.3389/fpsyt.2022.929413

39. Capriotti MR, Espil FM, Conelea CA, Woods DW. Environmental factors as potential determinants of premonitory urge severity in youth with Tourette syndrome. *J Obsessive Compuls Relat Disord* (2013) 2:37–42. doi: https://dx.doi.org/10.1016/j.jocrd.2012.10.004

40. Brandt V, Otte JH, Fremer C, Jakubovski E, Müller-Vahl K. Non-just-right experiences are more closely related to OCD than tics in Tourette patients. *Sci Rep* (2023) 13:19627. doi: 10.1038/s41598-023-37658-0

41. Kano Y, Fujio M, Kaji N, Matsuda N, Nonaka M, Kono T. Changes in Sensory Phenomena, Tics, Obsessive-Compulsive Symptoms, and Global Functioning of Tourette Syndrome: A Follow-Up After Four Years. *Front Psychiatry* (2020) 11:619. doi: 10.3389/fpsyt.2020.00619

42. Brandt VC, Niessen E, Ganos C, Kahl U, Bäumer T, Münchau A. Altered synaptic plasticity in Tourette’s syndrome and its relationship to motor skill learning. *PLoS One* (2014) 9:e98417-. doi: 10.1371/journal.pone.0098417

43. McGuire JF, McBride N, Piacentini J, Johnco C, Lewin AB, Murphy TK, Storch EA. The premonitory urge revisited: An individualized premonitory urge for tics scale. *J Psychiatr Res* (2016) 83:176–183. doi: 10.1016/j.jpsychires.2016.09.007

44. Larsh TR, Huddleston DA, Horn PS, Wu SW, Cecil KM, Jackson HS, Edden RAE, Mostofsky SH, Gilbert DL. From urges to tics in children with Tourette syndrome: associations with supplementary motor area GABA and right motor cortex physiology. *Cereb Cortex* (2022) doi: https://dx.doi.org/10.1093/cercor/bhac316

45. Batschelett MA, Huddleston DA, Crocetti D, Horn PS, Mostofsky SH, Gilbert DL. Biomarkers of tic severity in children with Tourette syndrome: Motor cortex inhibition measured with transcranial magnetic stimulation. *Dev Med Child Neurol* (2023) 65:1321–1331. doi: 10.1111/dmcn.15578

46. Che G, Ren W, McGuire JF, Li P, Zhao Z, Tian J, Zhang J, Zhang Y. Clinical evaluation of premonitory urges in children and adolescents using the Chinese version of Individualized Premonitory Urge for Tics Scale. *Front Psychiatry* (2023) 14:1224825. doi: 10.3389/fpsyt.2023.1224825

47. Li Y, Yan JJ, Cui YH. Clinical characteristics of pediatric patients with treatment-refractory Tourette syndrome: An evidence-based survey in a Chinese population. *World J Psychiatry* (2022) 12:958–969. doi: 10.5498/wjp.v12.i7.958

48. Sambrani T, Jakubovski E, Muller-Vahl KR. New insights into clinical characteristics of Gilles de la Tourette syndrome: Findings in 1032 patients from a single German center. *Front Neurosci* (2016) 10:415. doi: https://dx.doi.org/10.3389/fnins.2016.00415

49. Eapen V, Robertson MM. Are there distinct subtypes in Tourette syndrome? Pure-Tourette syndrome versus Tourette syndrome-plus, and simple versus complex tics. *Neuropsychiatr Dis Treat* (2015) 11:1431–1436. doi: https://dx.doi.org/10.2147/NDT.S72284

50. Kaczyńska J, Janik P. Tonic Tics in Gilles de la Tourette Syndrome. *Neuropediatrics* (2021) 52:370–376. doi: 10.1055/s-0040-1722689

51. Crossley E, Cavanna AE. Sensory phenomena: clinical correlates and impact on quality of life in adult patients with Tourette syndrome. *Psychiatry Res* (2013) 209:705–710. doi: 10.1016/j.psychres.2013.04.019

52. Brandt V, Essing J, Jakubovski E, Müller-Vahl K. Premonitory Urge and Tic Severity, Comorbidities, and Quality of Life in Chronic Tic Disorders. *Mov Disord Clin Pract* (2023) 10:922–932. doi: 10.1002/mdc3.13742

53. Langelage J, Verrel J, Friedrich J, Siekmann A, Schappert R, Bluschke A, Roessner V, Paulus T, Bäumer T, Frings C, et al. Urge-tic associations in children and adolescents with Tourette syndrome. *Sci Rep* (2022) 12: doi: 10.1038/s41598-022-19685-5

54. Silvestri PR, Chiarotti F, Giustini S, Cardona F. Alexithymia and tic disorders: a study on a sample of children and their mothers. *Eur Child Adolesc Psychiatry* (2019) 28:461–470. doi: https://dx.doi.org/10.1007/s00787-018-1209-x

55. Hagstrøm J, Spang KS, Vangkilde S, Maigaard K, Skov L, Pagsberg AK, Jepsen JRM, Plessen KJ. An observational study of emotion regulation in children with Tourette syndrome. *J Child Psychol Psychiatry* (2021) 62:790–797. doi: 10.1111/jcpp.13375

56. Cavanna AE, David K, Orth M, Robertson MM. Predictors during childhood of future health-related quality of life in adults with Gilles de la Tourette syndrome. *European Journal of Paediatric Neurology* (2012) 16:605–612. doi: https://dx.doi.org/10.1016/j.ejpn.2012.02.004

57. Kang NR, Kim HJ, Moon DS, Kwack YS. Effects of Group Comprehensive Behavioral Intervention for Tics in Children With Tourette’s Disorder and Chronic Tic Disorder. *Soa Chongsonyon Chongsin Uihak* (2022) 33:91–98. doi: 10.5765/jkacap.220013

58. Houghton DC, Capriotti MR, Scahill LD, Wilhelm S, Peterson AL, Walkup JT, Piacentini J, Woods DW. Investigating Habituation to Premonitory Urges in Behavior Therapy for Tic Disorders. *Behav Ther* (2017) 48:834–846. doi: https://dx.doi.org/10.1016/j.beth.2017.08.004

59. Deckersbach T, Chou T, Britton JC, Carlson LE, Reese HE, Siev J, Scahill L, Piacentini JC, Woods DW, Walkup JT, et al. Neural correlates of behavior therapy for Tourette’s disorder. *Psychiatry Res* (2014) 224:269–274. doi: 10.1016/j.pscychresns.2014.09.003

60. Nonaka M, Matsuda N, Kono T, Fujio M, Scahill L, Kano Y. Preliminary Study of Behavioral Therapy for Tourette Syndrome Patients in Japan. *Children’s Health Care* (2015) 44:293–306. doi: https://dx.doi.org/10.1080/02739615.2014.979922

61. Verdellen CW, Hoogduin CA, Kato BS, Keijsers GP, Cath DC, Hoijtink HB. Habituation of premonitory sensations during exposure and response prevention treatment in Tourette’s syndrome. *Behav Modif* (2008) 32:215–227. doi: 10.1177/0145445507309020

62. van de Griendt J, van den Berg NME, Verdellen CWJ, Cath DC, Verbraak M. Working Mechanisms of Exposure and Response Prevention in the Treatment of Tourette Syndrome and Tic Disorders Revisited: No Evidence for within-Session Habituation to Premonitory Urges. *J Clin Med* (2023) 12: doi: 10.3390/jcm12227087

63. Heijerman-Holtgrefe AP, Verdellen CWJ, van de Griendt JMTM, Beljaars LPL, Kan KJ, Cath D, Hoekstra PJ, Huyser C, Utens EMWJ. Tackle your Tics: pilot findings of a brief, intensive group-based exposure therapy program for children with tic disorders. *Eur Child Adolesc Psychiatry* (2021) 30:461–473. doi: https://dx.doi.org/10.1007/s00787-020-01532-5

64. Nissen JB, Parner ET, Thomsen PH. Predictors of therapeutic treatment outcome in adolescent chronic tic disorders. *BJPsych Open* (2019) 5:e74-. doi: 10.1192/bjo.2019.56

65. Sukhodolsky DG, Woods DW, Piacentini J, Wilhelm S, Peterson AL, Katsovich L, Dziura J, Walkup JT, Scahill L. Moderators and predictors of response to behavior therapy for tics in Tourette syndrome. *Neurology* (2017) 88:1029–1036. doi: https://dx.doi.org/10.1212/WNL.0000000000003710

66. Gilbert DL, Budman CL, Singer HS, Kurlan R, Chipkin RE. A D1 receptor antagonist, ecopipam, for treatment of tics in tourette syndrome. *Clin Neuropharmacol* (2014) 37:26–30. doi: https://dx.doi.org/10.1097/WNF.0000000000000017

67. Jankovic J, Jimenez-Shahed J, Brown LW. A randomised, double-blind, placebo-controlled study of topiramate in the treatment of Tourette syndrome. *J Neurol Neurosurg Psychiatry* (2010) 81:70–73. doi: https://dx.doi.org/10.1136/jnnp.2009.185348

68. McGuire JF, Ginder N, Ramsey K, Essoe JKY, Ricketts EJ, McCracken JT, Piacentini J. Optimizing behavior therapy for youth with Tourette’s disorder. *Neuropsychopharmacology* (2020) 45:2114–2119. doi: https://dx.doi.org/10.1038/s41386-020-0762-4

69. Jankovic J. Botulinum toxin in the treatment of dystonic tics. *Movement Disorders* (1994) 9:347–349. doi: http://dx.doi.org/10.1002/mds.870090315

70. Kwak CH, Hanna PA, Jankovic J. Botulinum toxin in the treatment of tics. *Arch Neurol* (2000) 57:1190–1193. doi: http://dx.doi.org/10.1001/archneur.57.8.1190

71. Marras C, Andrews D, Sime E, Lang AE. Botulinum toxin for simple motor tics: A randomized, double-blind, controlled clinical trial. *Neurology* (2001) 56:605–610. doi: http://dx.doi.org/10.1212/WNL.56.5.605

72. Kwak C, Dat Vuong K, Jankovic J. Premonitory sensory phenomenon in Tourette’s syndrome. *Mov Disord* (2003) 18:1530–1533. doi: 10.1002/mds.10618

73. Rath JJG, Tavy DLJ, Wertenbroek AAACM, van Woerkom TCAM, de Bruijn SFTM. Botulinum toxin type A in simple motor tics: Short-term and long-term treatment-effects. *Parkinsonism Relat Disord* (2010) 16:478–481. doi: https://dx.doi.org/10.1016/j.parkreldis.2009.11.011

74. Porta M, Maggioni G, Ottaviani F, Schindler A. Treatment of phonic tics in patients with Tourette’s syndrome using botulinum toxin type A. *Neurol Sci* (2004) 24:420–423. doi: 10.1007/s10072-003-0201-4

75. Di Lazzaro G, Magrinelli F, Ganos C, Bhatia KP. The Need to Tic. *Mov Disord Clin Pract* (2020) 7:863–864. doi: 10.1002/mdc3.13023

76. Muller-Vahl KR, Fremer C, Beals C, Ivkovic J, Loft H, Schindler C. Monoacylglycerol Lipase Inhibition in Tourette Syndrome: A 12-Week, Randomized, Controlled Study. *Movement Disorders* (2021) 36:2413–2418. doi: https://dx.doi.org/10.1002/mds.28681

77. Muller-Vahl KR, Fremer C, Beals C, Ivkovic J, Loft H, Schindler C. Endocannabinoid Modulation Using Monoacylglycerol Lipase Inhibition in Tourette Syndrome: A Phase 1 Randomized, Placebo-Controlled Study. *Pharmacopsychiatry* (2022) 55:148–156. doi: https://dx.doi.org/10.1055/a-1675-3494

78. Müller-Vahl KR, Kolbe H, Schneider U, Emrich HM. Cannabinoids: possible role in patho-physiology and therapy of Gilles de la Tourette syndrome. *Acta Psychiatr Scand* (1998) 98:502–506. doi: 10.1111/j.1600-0447.1998.tb10127.x

79. Anis S, Zalomek C, Korczyn AD, Rosenberg A, Giladi N, Gurevich T. Medical Cannabis for Gilles de la Tourette Syndrome: An Open-Label Prospective Study. *Behavioural Neurology* (2022) 2022:5141773. doi: 10.1155/2022/5141773

80. Abi-Jaoude E, Bhikram T, Parveen F, Levenbach J, Lafreniere-Roula M, Sandor P. A Double-Blind, Randomized, Controlled Crossover Trial of Cannabis in Adults with Tourette Syndrome. *Cannabis Cannabinoid Res* (2022) doi: 10.1089/can.2022.0091

81. Silver AA, Shytle RD, Sanberg PR. Clinical Experience With Transdermal Nicotine Patch in Tourette Syndrome. *CNS Spectr* (1999) 4:68–76. doi: 10.1017/S1092852900011408

82. Szejko N, Fremer C, Sühs KW, Macul Ferreira de Barros P, Müller-Vahl KR. Intravenous Immunoglobulin Treatment Did Not Improve Tics in a Patient With Gilles de la Tourette Syndrome and Intrathecal Antibody Synthesis. *Front Neurol* (2020) 11:110. doi: 10.3389/fneur.2020.00110

83. Szejko N, Jakubovski E, Fremer C, Muller-Vahl KR. Vaporized Cannabis Is Effective and Well-Tolerated in an Adolescent with Tourette Syndrome. *Med Cannabis Cannabinoids* (2019) 2:60–64. doi: https://dx.doi.org/10.1159/000496355

84. Bhikram T, Crawley A, Arnold P, Abi-Jaoude E, Sandor P. Neuroimaging the emotional modulation of urge inhibition in Tourette Syndrome. *Cortex* (2021) 135:341–351. doi: 10.1016/j.cortex.2020.10.010

85. Eddy CM, Cavanna AE, Rickards HE, Hansen PC. Temporo-parietal dysfunction in Tourette syndrome: Insights from an fMRI study of Theory of Mind. *J Psychiatr Res* (2016) 81:102–111. doi: 10.1016/j.jpsychires.2016.07.002

86. Eddy CM, Cavanna AE, Hansen PC. Empathy and aversion: the neural signature of mentalizing in Tourette syndrome. *Psychol Med* (2017) 47:507–517. doi: https://dx.doi.org/10.1017/S0033291716002725

87. Rae CL, Parkinson J, Betka S, Gouldvan Praag CD, Bouyagoub S, Polyanska L, Larsson DEO, Harrison NA, Garfinkel SN, Critchley HD. Amplified engagement of prefrontal cortex during control of voluntary action in Tourette syndrome. *Brain Commun* (2020) 2:18. doi: https://doi.org/10.1093/braincomms/fcaa199

88. Rae CL, Raykov P, Ambridge EM, Colling LJ, Gould van Praag CD, Bouyagoub S, Polanski L, Larsson DEO, Critchley HD. Elevated representational similarity of voluntary action and inhibition in Tourette syndrome. *Brain Commun* (2023) 5:fcad224-. doi: 10.1093/braincomms/fcad224

89. Tinaz S, Malone P, Hallett M, Horovitz SG. Role of the right dorsal anterior insula in the urge to tic in Tourette syndrome. *Mov Disord* (2015) 30:1190–1197. doi: 10.1002/mds.26230

90. Rae CL, Polyanska L, Gould van Praag CD, Parkinson J, Bouyagoub S, Nagai Y, Seth AK, Harrison NA, Garfinkel SN, Critchley HD. Face perception enhances insula and motor network reactivity in Tourette syndrome. *Brain* (2018) 141:3249–3261. doi: 10.1093/brain/awy254

91. Bhikram T, Arnold P, Crawley A, Abi-Jaoude E, Sandor P. The functional connectivity profile of tics and obsessive-compulsive symptoms in Tourette Syndrome. *J Psychiatr Res* (2020) 123:128–135. doi: 10.1016/j.jpsychires.2020.01.019

92. Tinaz S, Belluscio BA, Malone P, van der Veen JW, Hallett M, Horovitz SG. Role of the sensorimotor cortex in Tourette syndrome using multimodal imaging. *Hum Brain Mapp* (2014) 35:5834–5846. doi: 10.1002/hbm.22588

93. Loo SK, Miyakoshi M, Tung K, Lloyd E, Salgari G, Dillon A, Chang S, Piacentini J, Makeig S. Neural activation and connectivity during cued eye blinks in Chronic Tic Disorders. *Neuroimage Clin* (2019) 24:101956. doi: 10.1016/j.nicl.2019.101956

94. Niccolai V, Korczok S, Finis J, Jonas M, Thomalla G, Siebner HR, Müller-Vahl K, Münchau A, Schnitzler A, Biermann-Ruben K. A peek into premonitory urges in Tourette syndrome: Temporal evolution of neurophysiological oscillatory signatures. *Parkinsonism Relat Disord* (2019) 65:153–158. doi: 10.1016/j.parkreldis.2019.05.039

95. Ganos C, Kahl U, Brandt V, Schunke O, Baumer T, Thomalla G, Roessner V, Haggard P, Munchau A, Kuhn S. The neural correlates of tic inhibition in Gilles de la Tourette syndrome. *Movement Disorders* (2014) 29:S409-. doi: https://dx.doi.org/10.1002/mds.25914

96. He JL, Mikkelsen M, Huddleston DA, Crocetti D, Cecil KM, Singer HS, Edden RAE, Gilbert DL, Mostofsky SH, Puts NAJ. Frequency and Intensity of Premonitory Urges-to-Tic in Tourette Syndrome Is Associated With Supplementary Motor Area GABA+ Levels. *Movement Disorders* (2022) 37:563–573. doi: https://dx.doi.org/10.1002/mds.28868

97. Kanaan AS, Gerasch S, García-García I, Lampe L, Pampel A, Anwander A, Near J, Möller HE, Müller-Vahl K. Pathological glutamatergic neurotransmission in Gilles de la Tourette syndrome. *Brain* (2017) 140:218–234. doi: 10.1093/brain/aww285

98. Draganski B, Martino D, Cavanna AE, Hutton C, Orth M, Robertson MM, Critchley HD, Frackowiak RS. Multispectral brain morphometry in Tourette syndrome persisting into adulthood. *Brain* (2010) 133:3661–3675. doi: https://dx.doi.org/10.1093/brain/awq300

99. Sigurdsson HP, Pépés SE, Jackson GM, Draper A, Morgan PS, Jackson SR. Alterations in the microstructure of white matter in children and adolescents with Tourette syndrome measured using tract-based spatial statistics and probabilistic tractography. *Cortex* (2018) 104:75–89. doi: 10.1016/j.cortex.2018.04.004

100. Sigurdsson HP, Jackson SR, Jolley L, Mitchell E, Jackson GM. Alterations in cerebellar grey matter structure and covariance networks in young people with Tourette syndrome. *Cortex* (2020) 126:1–15. doi: 10.1016/j.cortex.2019.12.022

101. Sigurdsson HP, Jackson SR, Kim S, Dyke K, Jackson GM. A feasibility study for somatomotor cortical mapping in Tourette syndrome using neuronavigated transcranial magnetic stimulation. *Cortex* (2020) 129:175–187. doi: 10.1016/j.cortex.2020.04.014

102. Jackson SR, Sigurdsson HP, Dyke K, Condon M, Jackson GM. The role of the cingulate cortex in the generation of motor tics and the experience of the premonitory urge-to-tic in Tourette syndrome. *J Neuropsychol* (2021) 15:340–362. doi: 10.1111/jnp.12242

103. Hampson M, Tokoglu F, King RA, Constable RT, Leckman JF. Brain areas coactivating with motor cortex during chronic motor tics and intentional movements. *Biol Psychiatry* (2009) 65:594–599. doi: 10.1016/j.biopsych.2008.11.012

104. Bohlhalter S, Goldfine A, Matteson S, Garraux G, Hanakawa T, Kansaku K, Wurzman R, Hallett M. Neural correlates of tic generation in Tourette syndrome: an event-related functional MRI study. *Brain* (2006) 129:2029–2037. doi: 10.1093/brain/awl050

105. Duggal HS, Nizamie SH. Bereitschaftspotential in tic disorders: a preliminary observation. *Neurol India* (2002) 50:487–489.

106. Morera Maiquez B, Jackson GM, Jackson SR. Examining the neural antecedents of tics in Tourette syndrome using electroencephalography. *J Neuropsychol* (2022) 16:1–20. doi: 10.1111/jnp.12245

107. Zapparoli L, Devoto F, Mariano M, Seghezzi S, Servello D, Porta M, Paulesu E. Mapping Gilles de la Tourette syndrome through the distress and relief associated with tic-related behaviors: an fMRI study. *Transl Psychiatry* (2024) 14:7. doi: 10.1038/s41398-023-02711-z
